# Truncated titin is structurally integrated into the human dilated cardiomyopathic sarcomere

**DOI:** 10.1172/JCI169753

**Published:** 2024-01-16

**Authors:** Dalma Kellermayer, Hedvig Tordai, Balázs Kiss, György Török, Dániel M. Péter, Alex Ali Sayour, Miklós Pólos, István Hartyánszky, Bálint Szilveszter, Siegfried Labeit, Ambrus Gángó, Gábor Bedics, Csaba Bödör, Tamás Radovits, Béla Merkely, Miklós S.Z. Kellermayer

**Affiliations:** 1 Heart and Vascular Center,; 2 Department of Biophysics and Radiation Biology, and; 3 1st Department of Pathology and Experimental Cancer Research, Semmelweis University, Budapest, Hungary.; 4 DZHK Partnersite Mannheim-Heidelberg, Medical Faculty Mannheim, University of Heidelberg, Mannheim, Germany.

**Keywords:** Cardiology, Muscle Biology, Cardiovascular disease, Cytoskeleton, Muscle

## Abstract

Heterozygous (HET) truncating variant mutations in the TTN gene (TTNtvs), encoding the giant titin protein, are the most common genetic cause of dilated cardiomyopathy (DCM). However, the molecular mechanisms by which TTNtv mutations induce DCM are controversial. Here, we studied 127 clinically identified DCM human cardiac samples with next-generation sequencing (NGS), high-resolution gel electrophoresis, Western blot analysis, and super-resolution microscopy in order to dissect the structural and functional consequences of TTNtv mutations. The occurrence of TTNtv was found to be 15% in the DCM cohort. Truncated titin proteins matching, by molecular weight, the gene sequence predictions were detected in the majority of the TTNtv^+^ samples. Full-length titin was reduced in TTNtv^+^ compared with TTNtv^–^ samples. Proteomics analysis of washed myofibrils and stimulated emission depletion (STED) super-resolution microscopy of myocardial sarcomeres labeled with sequence-specific anti-titin antibodies revealed that truncated titin was structurally integrated into the sarcomere. Sarcomere length–dependent anti–titin epitope position, shape, and intensity analyses pointed at possible structural defects in the I/A junction and the M-band of TTNtv^+^ sarcomeres, which probably contribute, possibly via faulty mechanosensor function, to the development of manifest DCM.

## Abstract 2

Truncated mutants of titin are structurally incorporated in the sarcomere and cause axial disintegration, thereby leading to the pathogenesis of dilated cardiomyopathy.

## Graphical abstract

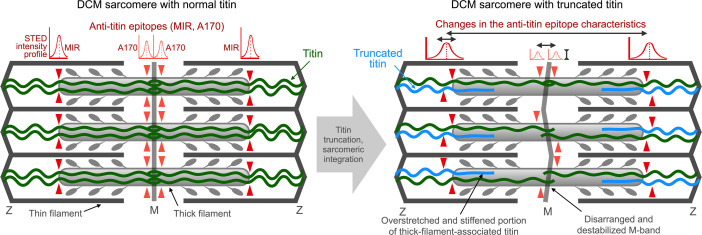

## Introduction

The giant myofilament titin is the third most abundant sarcomeric protein besides actin and myosin (1). A single titin molecule spans half of the sarcomere from the Z-disk to the M-line (2). Titin’s main function is to provide passive stiffness to striated muscle (3), but it also plays a prominent role in sarcomere development and assembly (4). Recent next-generation sequencing (NGS) data have shown that mutations in the TTN gene, which encodes titin, are associated with skeletal and cardiac myopathies (5–7). Among these, the most prevalent is dilated cardiomyopathy (DCM) (6). DCM is characterized by ventricular and atrial enlargement and reduced ventricular systolic function (8). The leading genetic cause of DCM is heterozygous (HET) truncating variant mutations in the TTN gene (TTNtvs), which account for approximately 15%–25% of familial cases (6, 9, 10). Most of the TTNtvs are nonsense and frameshift mutations, but splicing and copy number mutations occur as well (6, 11). TTNtvs are overrepresented in the A-band section of titin (6, 9) that consists of constitutively expressed exons. This section is thought to be functionally inextensible and acts as a molecular ruler for thick filament assembly (12). Interestingly, TTNtv mutations also appear in approximately 1% of the healthy population, mainly in nonconstitutive exons of the I-band, indicating a profuse splicing mechanism in titin’s I-band section (9).

The molecular mechanisms underlying TTNtv-induced DCM are still highly controversial. Multiple pathways have been proposed, including haploinsufficiency, the poison peptide mechanism, and perturbation of cardiac metabolism (10, 13, 14). Moreover, it has been suggested that the TTNtv mutation itself is often insufficient to induce a phenotype, but an additional factor, such as a second gene mutation, pregnancy, or an environmental stressor (e.g., alcohol, hypertonia, chemotherapy), is required to evoke clinical manifestation of the disease (15–21). Recent studies by Fomin et al. (22) and McAfee et al. (23) demonstrated, for the first time, the presence of truncated titin proteins in human cardiac samples of dilated cardiomyopathy as well as a reduction in total full-length titin. Furthermore, Fomin et al. showed that the truncated protein is not incorporated into the sarcomere but is accumulated in intracellular aggregates that act as toxic agents and impair protein quality control, supporting the poison peptide mechanism (22). By contrast, McAfee et al. suggested possible sarcomeric integration of the truncated protein (23). Furthermore, experiments on human induced pluripotent stem cell–derived cardiomyocytes (hiPSC-CMs) containing TTNtv showed impaired contractility relative to healthy controls (13, 24). Overall, these findings suggest that the truncated protein might be integrated into the sarcomere, supporting the existence of a poison peptide mechanism. However, the sarcomeric presence and arrangement of truncated titin has not yet been detected directly in the myocardium of patients with DCM.

Here, we investigated titin truncating variant mutations in a cohort of 127 patients with DCM. We analyzed the gene sequence of the samples to identify the location of titin truncation and investigated the protein expression profiles to reveal the corresponding protein products. The sarcomeric arrangement of truncated titin was explored with super-resolution microscopy on myocardial samples labeled with sequence-specific antibodies and exposed to mechanical stretch. We found that truncated titin is structurally integrated in the sarcomere and causes small, albeit probably functionally important, structural disturbances that are the possible contributors to the pathway toward DCM.

## Results

### Patient data and gene sequencing: identification of a TTNtv DCM cohort.

**Figure 1:**
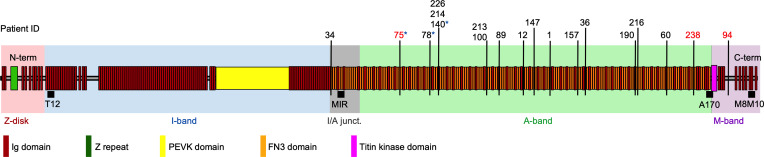
Titin domain structure and location of truncating variants. Layout of the human titin isoform IC (NCBI reference sequence NP_001254479) composed of 35,991 amino acids. NGS revealed 19 DCM samples with TTNtv, labeled according to the registry patient ID. The truncating variants were overrepresented in the A-band region of titin. Patient 34 had a truncating variant in the I/A junction (junct.), and patient 94 had the TTNtv in the M-band region. Red and starred numbers indicate samples used for myofibril protein composition and structural (STED microscopy) analyses, respectively. Black squares indicate the binding location of monoclonal antibodies used in the STED microscopy analyses. The T12 antibody binds to the I2–I3 titin domains (residues 2,174–2,437) (29, 30). The MIR, A170 and M8M10 antibodies bind to the I109–I112 (residues 15,968–16,348), A168–A170 (residues 33,496–33,784) and M8-M10 (residues 35,553–35,991) domains, respectively (see Supplementary Information and www.myomedix.com) (2, 31). term, terminal.

We screened 127 myocardial samples from explanted hearts of patients who were clinically identified as having DCM (Table 1, Supplemental Table 1, and Supplemental Table 2); supplemental material available online with this article; https://doi.org/10.1172/JCI169753DS1) for potentially pathogenic genetic variants using targeted exome sequencing (NGS). The DNA libraries were prepared so as to identify variants in 174 genes associated with inherited cardiac conditions (25). Fifty of the 174 genes are associated with DCM, whereas the remaining genes are implicated in inherited arrhythmias, other cardiomyopathies, aortopathies, and familial hypercholesterolemia, respectively. We identified 35,635 variants using the Genome Analysis Toolkit (GATK) pipeline (26), from which 13,815 were found in DCM-associated genes and 4,428 in the titin (TTN) gene (Supplemental Table 3). The variants were annotated by comparing with the Single Nucleotide Polymorphism Database (dbSNP), the Catalogue of Somatic Mutations in Cancer (COSMIC) database, and the ClinVar database. Based on the annotations and newly identified frameshift and nonsense variants, we found potentially pathogenic heterozygous mutations in 44 samples (Supplemental Table 3). In 35 samples (27.5%), the mutations were in DCM genes. We identified 19 TTNtv (15%), 4 lamin A (LMNA), 4 desmoplakin (DSP), 2 BAG cochaperone 3 (BAG3), and 1 each of the fukutin (FKTN), laminin Subunit Alpha 2 (LAMA2), myosin-binding protein C3 (MYBPC3), alpha heavy chain subunit of cardiac myosin (MYH6), beta heavy chain subunit of cardiac myosin (MYH7), phospholamban (PLN), RNA binding motif protein 20 (RBM20), and troponin I3 (TNNI3) variants (Supplemental Tables 3 and 4). Of the 19 pathogenic, likely pathogenic, and new heterozygous TTNtv mutations, we found that 8 were frameshift and 11 were nonsense mutations (Supplemental Table 4). All variants were located in the constitutively expressed I/A junction and the A- and M-band regions of titin (Figure 1 and Supplemental Table 5). In 2 TTNtv samples, we identified potentially pathogenic mutations in the Raf-1 Proto-Oncogene (RAF1) and transient receptor potential cation channel subfamily M member 4 (TRPM4) genes (Supplemental Table 4). On the basis of the NGS data, we divided our samples into DCM samples with (DCM^TTNtv+^, *n* = 19) or without (DCM^TTNtv–^, *n* = 108) titin truncation. We evaluated the echocardiographic data on the patients recorded prior to the transplantation to examine any TTNtv-associated phenotypes (Table 1 and Supplemental Table 2). Although more men had TTNtv mutations, similar to the finding of others (6), we did not find any genotype-associated phenotype severity in our DCM population. None of the functional parameters showed differences between the 2 groups.

### Protein analysis detects TTNtv subspecies and perturbed stoichiometries.

**Figure 2:**
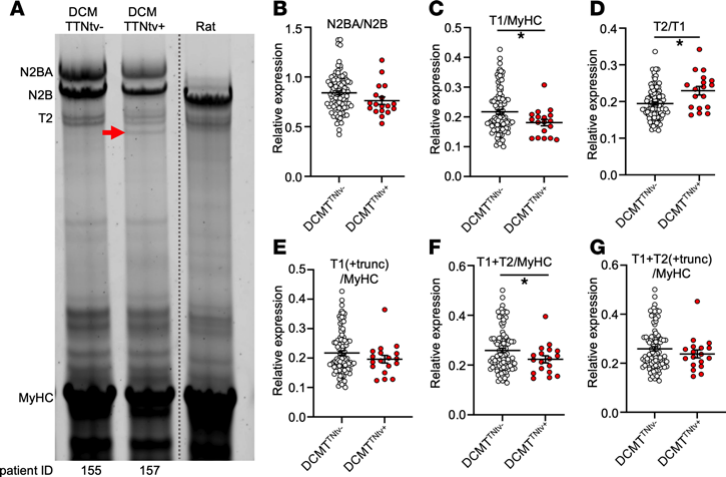
Titin isoform proteomics analysis. (**A**) Representative image of gel electrophoresis demonstrating titin N2BA and N2B isoforms, titin’s proteolytic degradation product (T2), and MyHC in TTNtv^–^ and TTNtv^+^ human myocardium and rat myocardium. The red arrow indicates the truncated protein. Linear contrast adjustment was applied to the original image for better visualization. The dotted line indicates where the different lanes of the same gel were digitally spliced together. (**B**) The N2BA/N2B ratio did not differ between TTNtv^+^ (*n* = 19) and TTNtv^–^ (*n* = 98) DCM groups. (**C**) Total titin (T1 = N2BA + N2B) normalized to MyHC was significantly reduced in the DCM^TTNtv+^ samples. (**D**) T2/T1 increased significantly in the TTNtv^+^ DCM samples. (**E**–**G**) Although T1/MyHC was reduced in the TTNtv^+^ DCM group, no differences were seen in the samples if the truncated (trunc) protein and T2 were added to T1. Values indicate the mean ± SEM. Samples were statistically compared by unpaired, 2-tailed Student’s *t* test; *P < 0.05. Grayscale saturation was avoided by measuring OD, and technical variability was negligible (see Supplemental Figure 2, C–F). For proteomics data statistics, see Supplemental Table 6.

Next, we analyzed the titin expression profiles of the myocardial samples with high-resolution gel electrophoresis (Figure 2). The N2BA/N2B titin isoform ratio was elevated in both the DCM^TTNtv–^ and DCM^TTNtv+^ groups compared with the physiological ratios obtained from data in the literature (27) (Figure 2B). The full-length titin to myosin heavy-chain ratio (T1/MyHC) was significantly decreased in the DCM^TTNtv+^ group (Figure 2C). The ratio of the T2 fragment (calpain-dependent proteolytic fragment that encompasses titin’s A-band section and a 100–200 kDa portion of its distal I-band section (28)) to full-length titin (T2/T1) was significantly increased in the DCM^TTNtv+^ samples (Figure 2D), suggesting that proteolytic activity may be increased in the DCM^TTNtv+^ myocardial tissue. Nevertheless, the (T1 + T2)/MyHC ratio was also significantly reduced in the DCM^TTNtv+^ samples (Figure 2F). We detected additional protein bands in the DCM^TTNtv+^ group (Figure 2A and Supplemental Figure 1), albeit not in all TTNtv^+^ samples. These proteins were observed on the gels at the most probable molecular weights calculated for the respective truncated titins from gene-sequencing data (Supplemental Table 5). Accordingly, we identified these bands as the protein products of the truncated titin genes. The average relative expression of the truncated proteins to full-length titin (T1) was 0.19. Notably, upon adding the truncated protein quantity to the respective T1 (Figure 2E), we observed no significant difference between the DCM^TTNtv+^ and DCM^TTNtv–^ samples. Furthermore, the integrated titin quantities (T1 + T2 + truncated titin) normalized to MyHC were essentially identical in DCM^TTNtv+^ and DCM^TTNtv–^ (Figure 2G and Supplemental Table 6).

**Figure 3:**
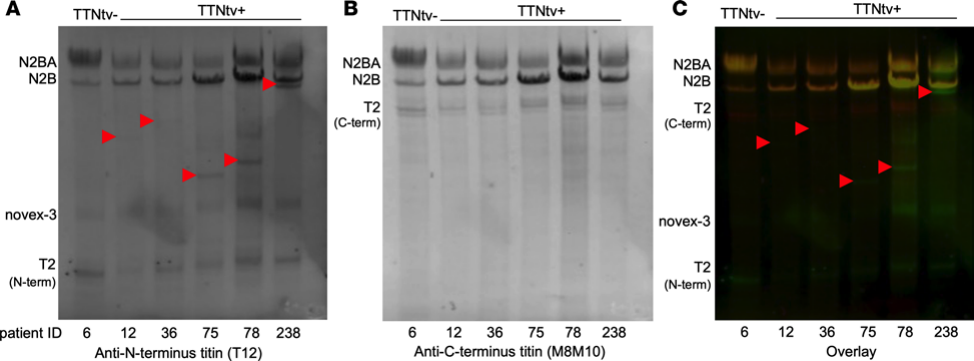
Differentiation of truncated titin from T2 by Western blot analysis. (**A**) Anti–N-terminus antibody (T2) detected truncated titin (red arrowheads), novex-3, and the N-terminus part of T2. (**B**) No signal was detected at the molecular level of the truncated proteins with the anti–C-terminus titin antibody M8M10, but it labeled the C-terminus part of T2. (**C**) The overlay image clearly differentiated the truncated titin from T2. Green tone indicates T12, red tone indicates M8M10 immunostaining, and red arrowheads point at truncated titin. The more complete Western blot analysis and its densitometric data on 18 of the DCM^TTNtv+^ samples are shown in Supplemental Figure 1 and Supplemental Table 16, respectively.

To experimentally test whether the additional protein bands were indeed truncated titins rather than the product of proteolysis (such as T2), we carried out Western blot analysis using sequence-specific antibodies targeting the C- and N-terminal regions of titin. The T12 antibody, which binds toward titin’s N-terminus (29, 30), labeled the additional protein bands but not T2 (Figure 3A and Supplemental Figure 1). By contrast, the M8M10 antibody, which binds near titin’s C-terminus (2, 31), labeled T2, but not the additional protein bands (Figure 3, B and C, and Supplemental Figure 1). Thus, we could differentiate the additional protein bands from T2, demonstrating that they contained titin’s N-terminal region and proving that they indeed corresponded to the protein products of the truncated titin genes.

**Figure 4:**
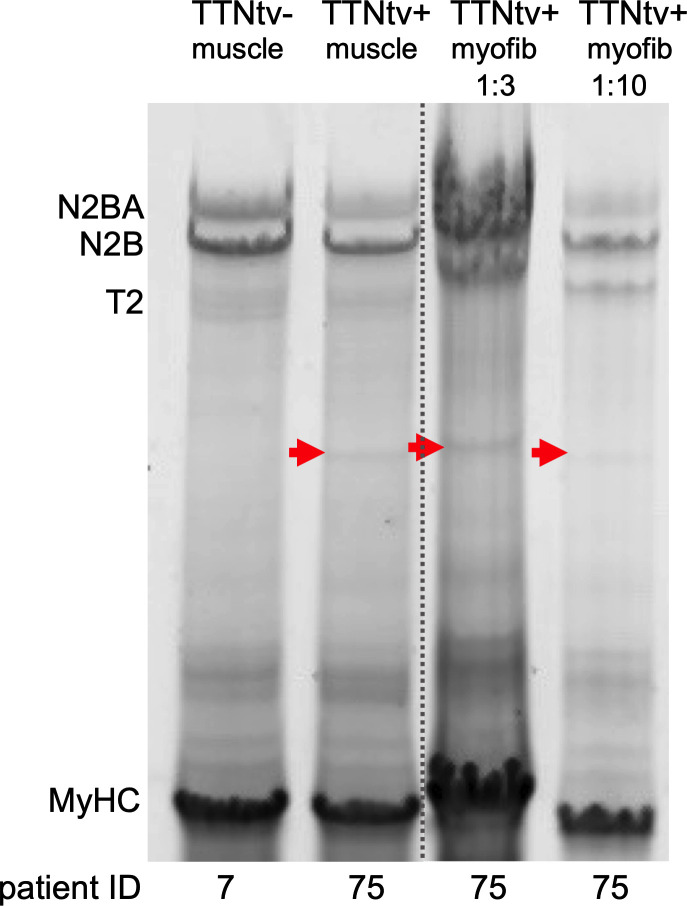
Skinned and washed myofibrils contain the truncated titin protein. SDS-PAGE electrophoretogram of washed myofibril (myofib) pellets solubilized in urea buffer at 1:3 and 1:10 ratios. In contrast to the TTNtv^–^ sample (patient 7), the TTNtv^+^ myofibril sample (patient 75) contained truncated titin, suggesting that the truncated protein was part of the sarcomere (see also Supplemental Figure 2A). In further support of this observation, the concentrated supernatants of the washed and centrifuged myofibril samples were devoid of detectable amounts of truncated titin (Supplemental Figure 2B). The gel image shown here is a spliced duplicate of the full gel shown in Supplemental Figure 2A. The dotted line indicates the splice. Red arrowheads point at truncated titin.

To investigate whether the truncated titins were incorporated into the sarcomere rather than present in the bulk of the sarcoplasm, we performed protein analysis of washed DCM^TTNtv+^ myofibrils (Figure 4 and Supplemental Figure 2, A–F). We were able to detect protein bands corresponding to the respective truncated titins in the gel electrophoretograms of washed DCM^TTNtv+^ myofibrils. Furthermore, the supernatants of the washed myofibril samples were devoid of truncated titin (Supplemental Figure 2B). Thus, we conclude that the truncated titin protein was incorporated into the cardiac muscle sarcomere.

### Super-resolution microscopy detects altered A/I junction and M-line widths.

**Figure 5:**
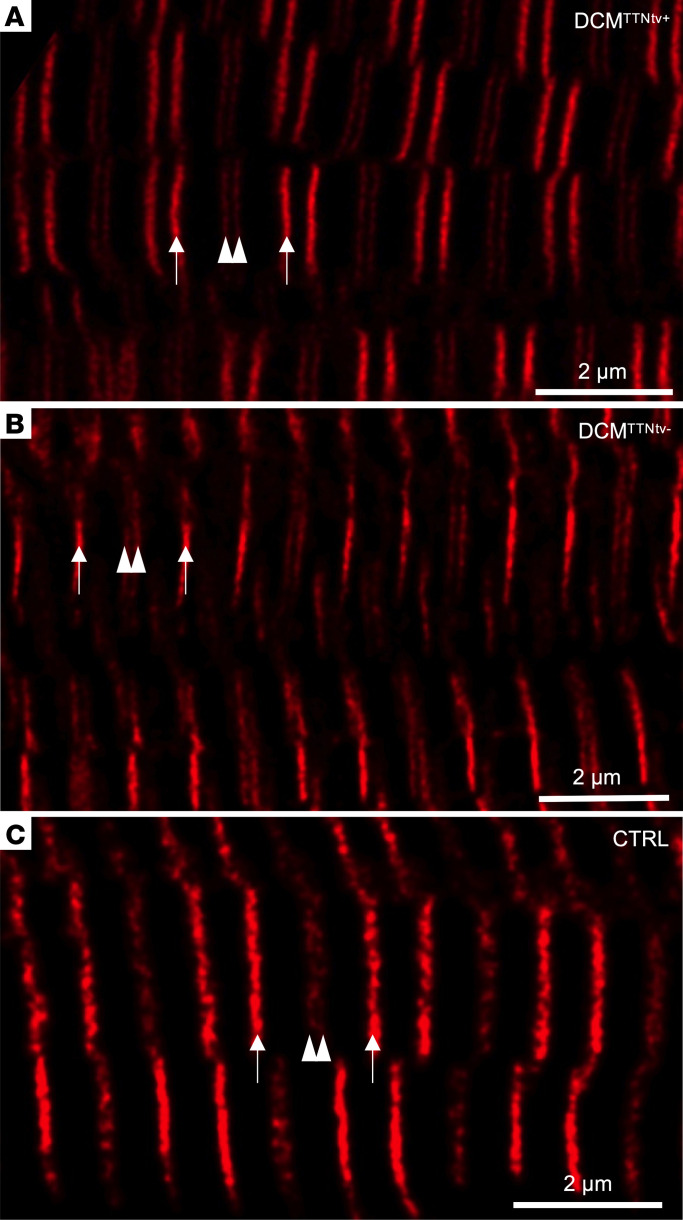
STED super-resolution microscopy of human DCM and normal control cardiac muscle samples. Representative STED images of DCM^TTNtv+^ (**A**), DCM^TTNtv–^ (**B**), and normal control (CTRL) (papillary muscle) (**C**) cardiac samples labeled with MIR and A170 antibodies. Arrows point to the MIR epitopes within 1 cardiac sarcomere, and arrowheads indicate the A170 epitopes near the M-line of the same sarcomere. Note that no gross structural alterations can be discerned in the DCM^TTNtv+^ sarcomeres. Scale bars: 2 μm.

**Figure 6:**
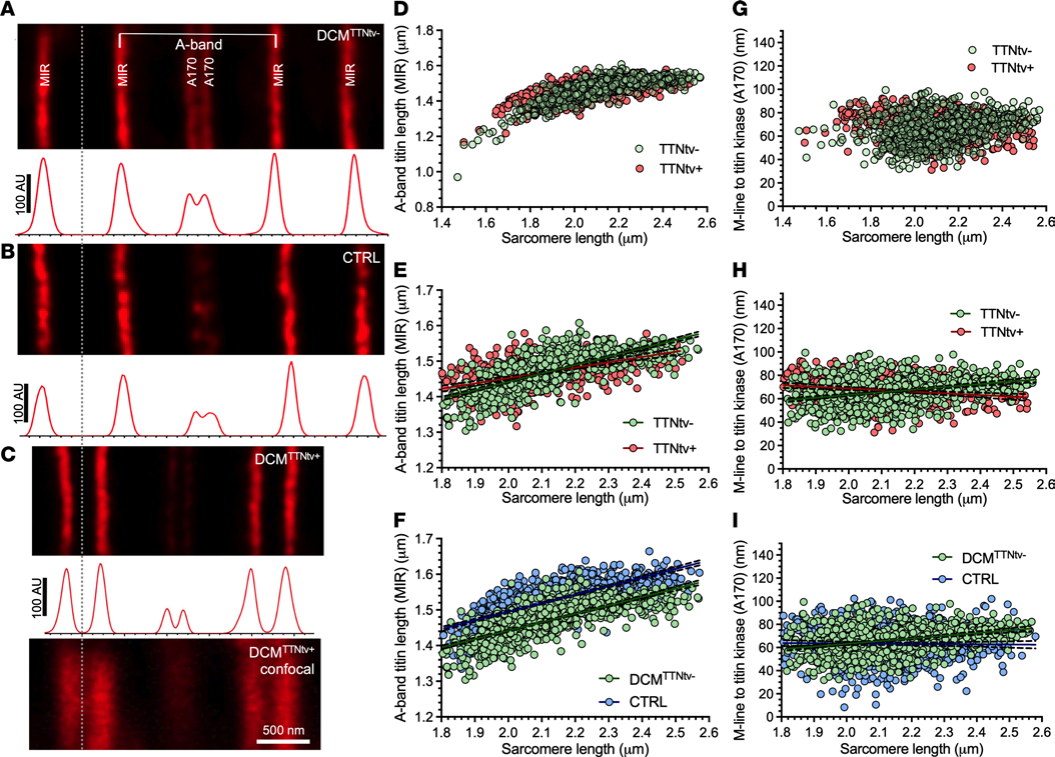
Titin epitope position and distance analysis. (**A**–**C**) Representative STED images and corresponding intensity plot profiles of DCM^TTNtv–^, negative control (human papillary muscle), and DCM^TTNtv+^ cardiac samples, respectively. Arrows and arrowheads point to the MIR and A170 titin epitopes, respectively. The plot profiles represent the fluorescence intensity distribution along the long axis of the sarcomere; the *x* axis therefore represents the distance (in nm), and the *y* axis indicates the fluorescence intensity (in AU). The sarcomere images are coaligned with respect to their left-side Z-lines, indicated by the vertical dotted line. Note the decreased A170 intensity in DCM^TTNtv+^ sarcomeres relative to that of the MIR epitope. The bottom panel in **C** is the respective confocal microscopic image, shown here to indicate the critical power of STED in resolving the separate A170 epitopes. Scale bar: 500 nm. (**D**) A-band titin length (measured as the distance between 2 consecutive MIR epitopes bounding an A170 epitope doublet) increased in both DCM^TTNtv–^ and DCM^TTNtv+^ cardiac fibers when the sarcomeres were passively stretched. (**E**) Linear regression analysis of the A-band titin length in the 1.8–2.6 μm sarcomere length range. There was a significant difference in the slopes of the linear fit (for statistics, see Supplemental Table 7). (**F**) Comparison of A-band titin lengths between the negative control and DCM^TTNtv–^ samples by linear regression. There was no significant difference in the slopes of the linear fit, but there was a significant, 44 nm difference in the *y* axis intercepts (for statistics, see Supplemental Table 8). (**G**) M-line–to–TK distance, calculated as the half value of the distance of 2 consecutive A170 epitopes, as a function of sarcomere length. (**H**) Linear regression analysis of the sarcomere length–dependent M-line–to–TK distance in the 1.8–2.6 μm sarcomere length range (for statistics, see Supplemental Table 9). (**I**) M-line–to–A170 epitope distance as a function of sarcomere length, for negative control and DCM^TTNtv–^ samples, compared with linear regression. The slope of the control data was not significantly different from zero (for statistics, see Supplemental Table 10). Three quasi-control (TTNtv^–^) and 3 TTNtv^+^ cardiac muscle samples (septum) were processed, and 3 skinned fiber bundles (technical replicates) used as a negative control were prepared from each papillary muscle sample. The fiber bundles were dissected from different locations of the cardiac muscle samples. At least 15 frozen sections were processed for STED imaging per technical replicate.

To explore whether and how truncated titin is structurally integrated into the sarcomere, we analyzed cardiac muscle samples labeled with sequence-specific anti-titin antibodies (MIR and A170, see Figure 1), using super-resolution stimulated emission depletion (STED) microscopy (Figure 5). We were unable to discern gross structural changes in the DCM^TTNtv+^ sarcomeres (Figure 5A) with respect to the DCM^TTNtv–^ (Figure 5B) and negative control samples (Figure 5C). The A170 doublet (separated by ~140 nm) could be resolved in all groups with STED (Figure 6, A–C), but not with confocal microscopy (Figure 6C bottom). Neither epitope doubling (Figure 6, A and C), nor significant intensity differences (data not shown) were found in the case of the MIR epitope which is present in both the full-length and truncated titin.

To investigate the structural integrity of the sarcomere-incorporated truncated titin, we carried out measurements on myocardial samples exposed to mechanical stretch. Epitope-to-epitope distance measurements revealed that the A-band titin length, measured as the distance between 2 consecutive MIR epitopes separated by an A170 epitope doublet (Figure 6A), increased in both DCM groups (Figure 6, D and E) and in the negative control (Figure 6F), as the fibers were passively stretched. Regression analysis of the A-band titin length in the 1.8–2.6 μm sarcomere length range revealed that, while the slopes were similar in the negative control and the DCM^TTNtv–^ sample (Figure 6F), the slope was significantly (*P* < 0.0001) reduced in the DCM^TTNtv+^ samples (Figure 6E). The MIR epitope was shifted toward the Z-disk at slack sarcomere length (1.8 μm) in the DCM^TTNtv+^ samples with respect to the DCM^TTNtv–^ samples (Figure 6E), although not as much as in the negative (Figure 6F). The MIR epitope position was less responsive to longitudinal stretch, as indicated by the significantly (*P* < 0.0001) lower A-band titin length values measured at longer sarcomere values. 

Measuring the distance between consecutive A170 epitopes allowed us to investigate the structural response of the titin kinase (TK) region to mechanical stretch (Figure 6, G–I). The TK region localized in the bare zone of the A-band approximately 70 nm from the M-line in both DCM^TTNtv–^ and DCM^TTNtv+^ sarcomeres, calculated as the half value of the distance of 2 consecutive A170 epitopes. In DCM^TTNtv–^, the M-line to TK distance increased by approximately 20 nm upon an increase in the sarcomere length from 1.8 to 2.6 μm (Figure 6H). By contrast, in the negative control, the M-line to TK distance remained essentially constant across this sarcomere length range (Figure 6I). Furthermore, to our surprise, the TK moved closer to the M-line in DCM^TTNtv+^ sarcomeres when the fibers were passively stretched, as indicated by the negative slope of the M-line to TK versus the sarcomere length function (Figure 6H). 

**Figure 7:**
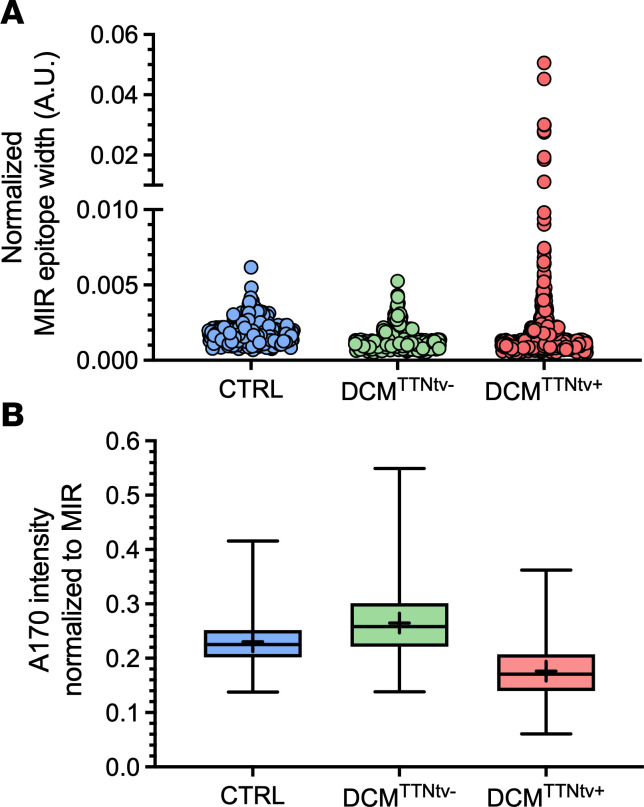
Titin epitope width and intensity analysis. (**A**) FWHM values normalized to the peak height of the MIR epitope of the negative control (papillary muscle), DCM^TTNtv–^, and DCM^TTNtv+^ sarcomeres between 1.8 and 2.6 μm. Note that the average sarcomere length values do not differ significantly between the 3 groups, hence, sampling across the investigated sarcomere length range is evenly distributed. For the data distribution across the entire sarcomere length range, see Supplemental Figure 4. Data were compared by 1-way ANOVA (see Supplemental Table 11). (**B**) A170 intensity normalized to the peak height of the neighboring MIR epitope within the half sarcomere for negative control (papillary muscle), DCM^TTNtv–^, and DCM^TTNtv+^ sarcomeres. The thick plus sign within the box shows the arithmetic mean, the line across the box represents the median, and the whiskers indicate the minimum and maximum values. Data were compared by 1-way ANOVA (see Supplemental Table 12).

To gain further insight into the possible arrangement of truncated titin in the sarcomere, we carried out measurements of epitope widths and intensities (Figure 7). The mean MIR epitope width was largest in DCM^TTNtv+^ sarcomeres in comparison with DCM^TTNtv-^ and the negative control and significantly greater than in DCM^TTNtv-^ samples (Figure 7A and Supplemental Table 11). Furthermore, the spread of the MIR width data points increased upon increasing the sarcomere length from 1.7 to 1.85 µm, then it declined upon further longitudinal sarcomere stretch (Supplemental Figure 4A). The relative intensity of the A170 epitope, normalized to the intensity of the MIR epitope of the same sarcomere, was significantly decreased in DCM^TTNtv+^ muscles (0.1765) compared to that in DCM^TTNtv-^ (0.2646), which is consistent with the missing epitope in truncated titin (compare Figure 6, A and C). The ratio of these intensities is 0.667, which is in good agreement with the proteomic ratio of the expressed truncated and full-length titins in the respective DCM^TTNtv+^ sample (Figure 7B). Notably, the normalized A170 intensity was also reduced in the negative control samples with respect to DCM^TTNtv-^ samples (Figure 6B and Figure 7B), which we attribute to variations in labeling efficiency. Therefore, we interpret the A170/MIR intensity ratios with caution. The A170 epitope width was significantly (*P* < 0.0001) greater in the DCM^TTNtv+^ and control sarcomeres than in the DCM^TTNtv–^ samples (Supplemental Figure 5).

## Discussion

Heterozygous TTNtvs are the most common genetic cause of familial DCM, accounting for 15%–25% of the cases (6, 9, 10). The pathomechanism by which titin mutations induce the cardiac phenotype are under extensive research (32). Although haploinsufficiency and a dominant negative effect have recently been suggested, on the basis of proteomics analyses (22, 23), the mechanistic links from the truncated titin protein to the sarcomeric structure and function remain highly controversial and debated (33). In order to dissect the role of titin in the pathogenesis of DCM, we performed NGS, high-resolution protein analysis, and super-resolved immunofluorescence microscopy combined with sarcomere extension on cardiac explant samples from a cohort of 127 patients with clinically diagnosed DCM.

We identified TTNtvs in 15% of our patient cohort, which is in accordance with prior NGS data (6, 9, 22). We uncovered additional, non-titin-related DCM and non-DCM-causing mutations in the samples (see Supplemental Tables 2 and 3). Clinical data revealed sex differences, as more men carried the truncating mutations than did women. However, the echocardiographic measurements revealed no differences between TTNtv^+^ versus TTNtv^–^ DCM patients (Table 1). It is important to note that the echocardiographic data were collected just prior to heart transplantation, by which time all of the patients had developed end-stage heart failure. Furthermore, there was a variation in the sample size of the echocardiographic data due to the heterogeneity in clinical profiling. Altogether, there were no substantial functional differences between the TTNtv^+^ and TTNtv^–^ DCM patients, which is in line with the recent study of McAfee et al. (23) (see also Supplemental Tables 1 and 2).

The evaluation of titin expression revealed increased titin N2BA/N2B ratios in all of the DCM samples compared with healthy donor heart data from the literature (27). We note here that we did not have any nonimplanted donor hearts for comparison and that the papillary muscle samples used as a negative control were so small that we could only use them for STED microscopy but not for electrophoresis. However, we observed no differences between the N2BA/N2B ratios in the 2 DCM groups, suggesting that the more compliant N2BA titin compensated for functional impairment in DCM despite the etiology of the disease (27). Similar to the findings of Fomin et al. (22) and McAfee et al. (23), we found that T1/MyHC was significantly (*P* < 0.05) decreased in the DCM^TTNtv+^ group, supporting the hypothesis that haploinsufficiency indeed contributed to the pathomechanism of TTNtv-induced DCM. In addition, we found significantly (*P* < 0.05) increased amounts of T2 in the TTNtv^+^ samples, which points to increased titin turnover related to the ubiquitin proteasome system, the pathogenic role of which has been suggested by Fomin et al. (22), and which needs to be clarified with further experiments. Importantly, however, we found that the integral titin amount, which included the full-length, truncated, and proteolysed proteins, was comparable in the DCM^TTNtv–^ and DCM^TTNtv+^ samples (Figure 2). We were able to identify truncated proteins in the majority (11 of the 19) of the TTNtv^+^ samples by gel electrophoresis. The truncated proteins were revealed on the gels at the molecular weight levels expected, based on the NGS data (Figure 2A and Supplemental Figure 1, and Supplemental Tables 5 and 6). The difference in titin expression in DCM^TTNtv–^ and DCM^TTNtv+^ samples, calculated as the T1/MyHC ratio (Figure 2C), was alleviated if the truncated proteins were taken into account in calculating total titin in the DCM^TTNtv+^ samples (Figure 2, E and G). Because the expressed amount of full-length and truncated titin proteins together was not significantly reduced in DCM^TTNtv+^, the specific truncated sections of the titin molecule may have harbored important functionality. Thus, the structural and mechanical consequences of the truncated titin protein must be investigated in detail.

Using Western blot analysis, we were able to establish that the additional protein bands, identified putatively as the protein products of the truncated titin gene, were indeed truncated titins rather than further degradation products of T2 (Figure 3 and Supplemental Figure 1, and Supplemental Table 16). Although the M8M10 antibody, which targets titin near its C-terminus, labeled T1 and T2 exclusively (Figure 3B and Supplemental Figure 1 lower panel), the T12 antibody, which targets titin near its N-terminus, labeled T1 and all the additional protein fragments as well (Figure 3A and Supplemental Figure 1, upper panel). Thus, the additional protein bands indeed corresponded to the protein products of the truncated titin genes. We note here that we could not detect truncated titins in all of the DCM^TTNtv+^ samples (8 of 19) and that some low-quantity truncated titin protein could be detected only by Western blotting (Supplemental Figure 1 and Supplemental Table 16). Conceivably, truncated protein was not produced in all cases (10), or in quantities so low that it remained below the detection threshold of our technique. Moreover, the quantity of the truncated proteins was uneven in spite of similar penetrance of TTNtv. Understanding how low expression of TTNtv leads to disease manifestation requires extensive further research, particularly because we do not know the exact mechanisms that lead to pathology even in cases in which the truncated protein is clearly identified. We speculate that the titin interactome (34) is sensitive to the partial loss of titin, even in amounts too small to be detected with the current proteomics methods.

To explore whether the truncated titin was incorporated into the sarcomere, we first analyzed the protein composition of washed myofibrils that were devoid of the sarcoplasm. Electrophoretic analysis of skinned and washed DCM^TTNtv+^ myofibril samples revealed the presence of the respective truncated titin in the myofibrillar fraction (Figure 4 and Supplemental Figure 2A) but not in the concentrated supernatant (Supplemental Figure 2B). The results support the findings of Fomin et al. and McAfee et al. and suggest a poison peptide mechanism (22, 23). Fomin et al. hypothesized that the truncated proteins are accumulated as intracellular aggregates (22). The study by McAfee et al. revealed TTNtv variants in sarcomere-containing cellular fractions, suggesting that the truncated titin is incorporated into the sarcomere (23). However, they could not rule out the possibility that the truncated titins are solely present as nonsarcomeric aggregates (23); therefore, whether the truncated titin molecule is structurally and mechanically integrated into the sarcomere remained a puzzling question.

To uncover the arrangement of truncated titin in the slack and extended sarcomere, we performed STED super-resolution microscopy on negative control, DCM^TTNtv–^, and DCM^TTNtv+^ myocardial tissue samples exposed to mechanical stretch and labeled with sequence-specific anti-titin antibodies (Figures 5–7, Supplemental Figures 3–6, and Supplemental Tables 11, 13–15). It is important to note that, because the truncated titin molecules do not carry epitopes that are unique with respect to the full-length molecule, the immunofluorescence microscopic results provided only indirect evidence of the truncated titin’s sarcomeric behavior. Since both the full-length and truncated titins are likely present in the sarcomere because of the heterozygous nature of TTNtv, truncated titin behavior may be inferred from the number, location, intensity and spatial width of the antibody epitope label signals within the sarcomere. We used 2 anti-titin antibodies to monitor different regions of titin. MIR labels the I/A junction of titin, and A170 is localized at the TK region, at the edge of the bare zone of the A-band. Considering that TTNtv was overrepresented in the A-band region, MIR and A170 labeled all and none of the studied DCM^TTNtv+^ samples, respectively (Figure 1). Such differentiated labeling allowed us to gain precise insight into the sarcomeric behavior of the truncated titin molecules.

We observed no gross structural disturbance in the DCM^TTNtv+^ sarcomeres (Figure 5A) in comparison with DCM^TTNtv–^ (Figure 5B) and negative control (Figure 5C) sarcomeres, and we could not detect fluorescence signal in unexpected locations, such as on the surface of the myofibrils or in between the expected epitope locations. In fact, given the overall across-the-sarcomere appearance of both the MIR and A170 epitopes, the myofilaments were in precise registry. Notably, sarcomeric structure was homogenous across the microscopic fields of view, indicating that the truncated titin molecules were distributed homogenously throughout the sample, rather than being confined to distinct sarcomeres that would appear as structural mosaicism. We successfully resolved the A170 epitope doublet, with an average separation distance of approximately 140 nm, owing to the high resolution of STED microscopy, which was tested to be approximately 40 nm in our instrument. It was important to be able to resolve the A170 epitope doublet so as to alleviate confounding of intensity measurements and to uncover the behavior of the TK region. The average intensity of the A170 epitope was reduced in the TTNtv^+^ samples by 23% and 33% with respect to the normal control and TTNtv^–^ samples, respectively (Figure 7B and Supplemental Table 12), whereas that of the MIR epitope remained unchanged, indicating that the truncated titin was indeed incorporated into the sarcomere, and supporting our protein analysis results for washed myofibrils. Why the A170 epitope intensity was smaller in the normal control than in the TTNtv^–^ samples needs further investigation, but it may be associated with tissue specificities (papillary muscle) that affect antibody labeling efficiency. Finally, the lack of MIR epitope doubling suggests that the truncated titin was not only incorporated into the sarcomere but structurally integrated similarly to the full-length form.

The precise epitope localization made possible by STED microscopy allowed us to study the structural rearrangements of titin in the negative control, DCM^TTNtv–^, and DCM^TTNtv+^ sarcomeres exposed to a partial functional assay in the form of mechanical stretch (Figures 6 and 7, Supplemental Figures 3–6, and Supplemental Tables 11, 13–15). The A-band titin length, measured as the MIR-to-MIR distance (Figure 6A) increased in all groups upon passive stretch (Figure 6, D–F), which supports earlier notions that the A-band section of titin is genuinely extensible (35). Interestingly, however, regression analysis revealed a significantly reduced slope in the TTNtv^+^ samples across the 1.8–2.6 μm sarcomere range, which points to a reduced A-band extensibility in the DCM^TTNtv+^ sarcomere (for statistical comparison, see Supplemental Table 7). Notably, the MIR epitope was shifted toward the Z-disk in the DCM^TTNtv+^ sarcomere at slack (1.8 μm) (Figure 6E), suggesting that A-band titin was more extended or, vice versa, that the I-band titin was more contracted on average (Supplemental Figure 3D) than in DCM^TTNtv–^ sarcomeres. Notably, the A-band titin width at slack sarcomere length was smaller in both DCM^TTNtv–^ and DCM^TTNtv+^ sarcomeres than in the negative control sarcomeres (Figure 6F), suggesting that titin’s sarcomeric arrangement as affected in DCM, irrespective of the presence of truncation. The differences in sarcomere length–dependent MIR-to-MIR epitope distance behavior were coupled with a significantly increased MIR epitope width in the DCM^TTNtv+^ sarcomeres with respect to both the normal control and DCM^TTNtv–^ samples (Figure 7A and Supplemental Table 11), indicating that there was a slight disarrangement among the titin molecules in spite of the gross alignment (i.e., there was no MIR epitope doubling). Presumably, the I-band section of the truncated titin molecules had become more contracted, owing to the a priori weaker A-band attachment, which resulted in the widening of the MIR epitope. Notably, the average MIR epitope width in the DCM^TTNtv+^ sarcomeres decreased with increasing sarcomere length (Supplemental Figure 4), suggesting that the axial titin disarrangement may have been reduced by mechanical stretch.

The response of the TK region to sarcomere stretch was quite different in the DCM^TTNtv–^ versus DCM^TTNtv+^ and negative control sarcomeres (Figure 6, H and I). While the M-line–to–TK distance remained constant in the negative control and increased with sarcomere length in DCM^TTNtv–^ sarcomeres, it progressively decreased in DCM^TTNtv+^ sarcomeres. The increase in the M-line–to–TK distance in DCM^TTNtv–^ sarcomeres provides direct evidence that the TK indeed responded, probably by in situ partial unfolding (36), to mechanical stretch. Notably, the extensibility of the TK region in DCM^TTNtv+^ sarcomeres was more than twice as large as in the entire A-band section of titin: ~20 nm extension/~60 nm initial length (Figure 6H) versus ~200 nm extension/~1,400 nm initial length (Figure 6E), which points to a differential control of titin elasticity or conformation along the thick filament. The lack of detectable TK extension in the normal control might be attributable to tissue specificity (papillary muscle) that needs to be explored further. The reduction in M-line–to–TK distance in DCM^TTNtv+^ is puzzling, considering that an apparent contraction in the TK region upon sarcomere stretch was unexpected. The controversial TK region behavior was coupled with a significantly (*P* < 0.0001) increased A170 epitope width in comparison with DCM^TTNtv–^ sarcomeres (Supplemental Figure 5), indicating that there was structural disarrangement among the fewer full-length titin molecules in the bare zone of the DCM^TTNtv+^ sarcomere. We note that the A170 epitope width was even greater in the normal control (Supplemental Figure 5), which may also be due to tissue specificities. It is also notable that we observed a patient-dependent variation in the mean M-line–to–TK distance (Supplemental Figure 6, C and D) that showed a positive correlation with the truncated titin/T1 titin ratio (Supplemental Figure 6E), suggesting that the ratio of the number of full- versus partial-length titin molecules in the sarcomere had a functional effect on the TK region.

**Figure 8:**
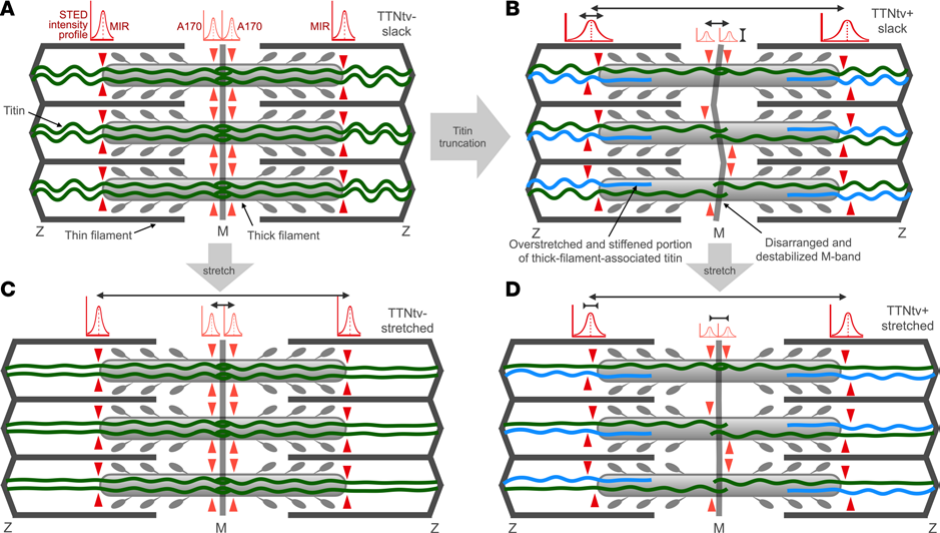
Schematic model of the structural and mechanical changes in the cardiac sarcomere caused by the presence of truncated titin. Gaussian functions above the sarcomere schemes indicate the intensity profiles of the anti-titin epitopes used in this work. The red arrowheads indicate the epitope positions in the respective titin molecules. The black double-headed arrows indicate the changes in measured parameters. (**A**) TTNtv^_^ sarcomere at slack. Full-length titin molecules (6 per half thick filament, shown as green lines) are present. The anti-titin epitopes are in register, and the corresponding STED profiles are of high intensity and have a narrow width. (**B**) TTNtv^+^ sarcomere at slack. Truncated titin (shown as blue lines) is incorporated into the sarcomere (3 per half thick filament on average, presumably randomly distributed). Because the anchorage of TTNtv in the A-band is compromised, the molecules are pulled slightly toward the Z-line (hence, the A-band titin length is increased). The MIR epitopes are slightly out of register, resulting in an increase in the STED epitope profile width. Because only about half of the titins contribute to the A170 labeling, the A170 epitope intensity is reduced. The M-line–to–A170 distance is slightly increased (by ~10 nm), suggesting that the M-band region is disarranged (indicated by a crooked M-band). (**C**) TTNtv^–^ sarcomere after stretch. The apparent A-band titin length is increased (by ~236 nm per every micrometer increment of sarcomere length), but the MIR epitopes remain in register. The M-band–to–A170 epitope distance is increased upon sarcomere stretch, which is most likely due to structural changes in the TK domain related to its mechanosensory function (ref. 36). (**D**) TTNtv^+^ sarcomere after stretch. The apparent A-band titin length is increased, but to a smaller degree than in the TTNtv^–^ sarcomere. The MIR epitopes on the normal and truncated titin molecules approach each other, resulting in a relative narrowing of the STED intensity profile (see Supplemental Figure 4A). The M-line–to–A170 epitope distance becomes reduced upon sarcomere stretch due to a mechanically driven ordering, indicated by a straightened M-band.

We propose the following model to explain our complex and somewhat puzzling observations (Figure 8). In contrast to the normal sarcomere (Figure 8A), in DCM^TTNtv+^ sarcomeres, different numbers of full-length and truncated titin molecules are integrated into the sarcomere, the ratio of which is controlled by the penetrance of the genetic condition. Because the anchorage of TTNtv in the A-band is compromised, the molecules are pulled slightly toward the Z-line by their intact I-band sections. Therefore, in the slack DCM^TTNtv+^ sarcomere, the A-band titin length is increased, and, vice versa, the I-band titin length is reduced. However, this disposition (and hence the titin disarrangement) is slight, due probably to a prestretched state of the A-band section of the truncated titin that enhances its binding within the A-band. The MIR epitopes are slightly out of register, resulting in an increase in the STED epitope profile width. Because TTNtv lacks a good portion of its A-band section and its entire M-band section, only about half of the titins contribute to the A170 signal, hence, the A170 epitope intensity is reduced. The reduced number of titins in the bare zone and M-band likely results in structural disarrangement and weakening, leading to an increase in both the M-line–to–A170 distance (by ~10 nm) and the width of the A170 epitope (Figure 8B). This pathological disarrangement is indicated in the figure, albeit in an exaggerated way, by a crooked M-band. Upon stretch (Figure 8, C and D), the apparent A-band titin length is increased, but to a smaller degree than in the DCM^TTNtv–^ sarcomere, given the prestretched and stabilized nature of the truncated titin molecules. Accordingly, the MIR epitopes on the normal and truncated titin molecules approach each other, resulting in a relative narrowing of the STED intensity profile. The M-line–to–A170 epitope distance becomes reduced upon sarcomere stretch, which is a paradoxical phenomenon due, conceivably, to a mechanically driven ordering in the M-band. In principle, the faulty mechanosensor function of the M-band revealed here may be a major pathway leading to manifest DCM. Although some elements of our proposition, such as the prestretched and stabilized A-band portion of the truncated titin and the structurally disarranged M-band, are hypothetical and need further exploration, the model is consistent with our experimental data and provides testable predictions.

In conclusion, our results provide strong support for the notion that titin truncating variants are a major cause of familial DCM. Truncated titin molecules are incorporated and integrated into the sarcomere and likely cause small but functionally important internal structural and mechanical perturbations. The compensatory effects in the I/A junction and the faulty mechanosensor function in the M-band region of titin probably play a substantial role in the pathway toward DCM.

## Methods

### Sample collection and handling.

Human myocardial tissue samples were obtained from the Transplantation Biobank of the Heart and Vascular Center at Semmelweis University in Budapest, Hungary. Myocardial septum samples were collected from 127 patients with clinically identified end-stage DCM, who were undergoing orthotopic heart transplantation (HTx). The samples were surgically dissected from the explanted, diseased hearts of the recipients. The septum samples were immediately snap-frozen in liquid nitrogen under sterile conditions and stored at –80°C for further measurements and analyses. Echocardiographic data recorded prior to surgery were obtained from our Transplantation Biobank database. The sample ID numbers shown in the images are the patients’ ID numbers from the Heart Transplantation registry. As a non-DCM negative control, left ventricular papillary muscle samples were collected in 3 separate open-heart surgeries. Given the extremely small size of these samples, they were used only for structural (STED microscopy) measurements.

### Gene sequencing.

Genomic DNA was isolated from 25 mg frozen septum samples using the QIAamp DNA Mini Kit (QIAGEN) according to the manufacturer’s recommendation. NGS of the purified DNA (50 ng) was performed using the Illumina TruSight Cardio library preparation kit (25), with the libraries sequenced on an Illumina MiSeq instrument. Quality control of the raw *fastq* files was performed with the FastQC (version 0.11.9) and MultiQC (version 1.9) algorithms (37, 38). Further bioinformatic analyses were carried out with the Broad Institute’s Genome Analysis Toolkit (GATK) Best Practices of Germline Short Variant Discovery (26). Sequence alignment to the hg19 genome version was performed using the Burrows-Wheeler Aligner (BWA) (version 0.7.17), then the mapped reads underwent duplicate marking (GATK-MarkDuplicatesSpark tool) and Base Quality Score Recalibration (GATK-BQSR tool) (39). The variant calling step was performed for every sample (GATK-Haplotype Caller). The GVCF files produced for every analyzed sample were consolidated into a single file (GATK-GenomicsDBImport) in order to perform joint genotyping (GATK-GenotypeGVCF). This cohort-wide view facilitated highly sensitive detection of variants even at difficult genomic sites. Final filtering was performed by hardfiltering with the following parameters: MIN_QD=2MAX_FS=60. The filtered variants were annotated with dbSNP (version human_9606_b151), COSMIC (version 92), Ensembl-Variant Effect Predictor, and ClinVar (version 2020.07.17) databases.

### Protein solubilization.

Pieces of the myocardial septum samples (10–15 mg) were homogenized in glass Kontes Dounce tissue grinders under liquid nitrogen. After 20 minutes of incubation at –20°C, the samples were solubilized at 60°C for 15 minutes in 50% urea buffer (8 M urea, 2 M thiourea, 50 mM Tris-HCl, 75 mM DTT, 3% SDS, and 0.03% bromophenol blue, pH 6.8) and 50% glycerol containing protease inhibitors (0.04 mM E64, 0.16 mM leupeptin, and 0.2 mM PMSF). All solubilized samples were centrifuged at 16, 000 x *g* for 5 minutes, aliquoted, flash-frozen in liquid nitrogen, and stored at –80°C (40).

### Titin isoform analysis.

Titin expression levels were determined with 1% SDS–agarose gel electrophoresis (41), performed at 16 mA/gel for 3.5 hours. Subsequently, the gels were stained overnight with SYPRO Ruby Protein Gel Stain (Thermo Fischer Scientific) and then digitized with a Typhoon laser scanner (Amersham BioSciences). ImageJ (NIH) was used to analyze the OD of the titin bands. The relative titin isoform ratio (N2BA/N2B) was calculated from the integrated band densities. The relative content of full-length titin (T1) that included N2BA and N2B was normalized to MyHC. T2 (titin’s proteolytic degradation product) was normalized to T1. Truncated proteins detected on the gels were normalized to T1.

### Detection of truncated titin products.

To determine whether the suspected band visible on the gel was indeed a truncated titin product, we performed Western blot analysis. The samples were separated on a 0.8% SDS-agarose gel and then transferred onto a PVDF membrane (Hybond-LFP, Amersham BioSciences) using a semi-dry blotter (Trans-Blot Cell, Bio-Rad). To differentiate the truncated product from T2, we used 2 antibodies, each detecting the terminal regions of titin. The blots were probed with anti-T12 (binds near titin’s N-terminus, provided by Dieter O. Fürst, University of Bonn, Bonn, Germany; dilution 1:1,000) (29) and anti-M8M10 (TTN-9, Myomedix, dilution 1:1,000; see Supplemental Information) primary antibodies overnight at 4°C, followed by secondary CyDye-conjugated antibodies (Amersham BioSciences). Subsequently, the blots were digitized with a Typhoon laser scanner. Relative expression levels of the proteins were analyzed using ImageJ.

### Preparation of myofibril suspension.

The myofibril suspension was prepared as previously described (42). Briefly, 2 mL ice-cold permeabilization solution (10 mM Tris [pH 7.1], 132 mM NaCl, 5 mM KCl, 1 mM MgCl_2_, 5 mM EGTA, 5 mM DTT, 10 mM NaN_3_, 20 mM 2,3-butanedione-monoxime [BDM], and 1% Triton X-100) containing protease inhibitors (0.04 mM E64, 0.16 mM leupeptin, and 0.2 mM PMSF) was added to previously frozen small pieces of septum samples (total weight, 15 mg). Next, the samples were incubated on a 360° rotating shaker for 3 hours at 4°C. The permeabilized samples were subsequently rinsed in washing solution (same as permeabilization solution, but without Triton X-100 and BDM) for 15 minutes. To prepare the myofibril suspension, the samples were transferred into 1 mL ice-cold fresh washing solution and homogenized with an MT-30K Handheld Homogenizer for 10–15 seconds at 27,000 rpm (Hangzhou Miu Instruments). The myofibrils were pelleted by applying low-speed centrifugation followed by resuspension and washing. The centrifugation and washing steps were performed at least twice. Subsequently, the pellet was solubilized in 50% urea buffer and 50% glycerol with inhibitors (1:1) and was incubated for 15 minutes at 60°C. Additional 1:3 and 1:10 dilutions of the myofibril suspension/urea buffer solution were prepared. The solubilized myofibril samples were separated on 1% SDS–agarose gels.

### Super-resolution microscopy.

Pieces of flash-frozen DCM^TTNtv–^ (*n* = 3) and DCM^TTNtv+^ (*n* = 3) left ventricular cardiac muscle were dissected in relaxing solution (40 mM BES, 10 mM EGTA, 6.56 mM MgCl_2_, 5.88 mM Na-ATP, 1 mM DTT, 46.35 mM K-propionate, and 15 mM creatine phosphate, pH 7.0) containing 1% (w/v) Triton X-100 and protease inhibitors (0.1 mM E64, 0.47 mM leupeptin, and 0.25 mM PMSF). The dissected cardiac muscle pieces were skinned overnight at 4°C in relaxing solution containing 1% Triton X-100 and protease inhibitors, washed thoroughly for at least 5 hours with relaxing solution, and then used immediately for experiments. Myofibril bundles were prepared and stretched from the slack length to different degrees (~40%–70%) and then fixed with 4% (v/v) formaldehyde diluted with phosphate buffer at neutral pH. Fixed bundles were embedded in OCT compound and frozen immediately in 2-methylbutane precooled by liquid nitrogen. Cryosections (8 μm thick) were then cut with a Microm Cryo Star HM 560 cryostat (Thermo Fisher Scientific) and mounted onto microscope slides (Superfrost UltraPlus, Thermo Fisher Scientific). Tissue sections were permeabilized in 0.2% Triton X-100 in PBS for 20 minutes at room temperature, blocked with 2% BSA and 1% normal donkey serum in PBS for 1 hour at 4°C, and incubated overnight at 4°C with primary antibodies and phalloidin diluted in blocking solution. The primary antibodies included rabbit polyclonal anti–titin MIR (TTN-7; www.myomedix.com, 0.4 μg/mL, 1:300 dilution), rabbit polyclonal anti–titin A170 (TTN-8; www.myomedix.com, 0.7 μg/mL, 1:250 dilution), and Alexa Fluor 488–conjugated phalloidin (Invitrogen, Thermo Fisher Scientific, A-12379, 66 μmol/L, 1:500 dilution). Sections were then washed twice for 30 minutes with PBS and incubated with Abberior STAR580 goat anti–rabbit IgG (Abberior) (1:250 dilution) and Alexa Fluor 488–conjugated phalloidin (Invitrogen, Thermo Fisher Scientific, A-12379, 66 μmol/L, 1:500 dilution). The sections were then washed twice for 15 minutes with PBS and covered with number 1.5H high precision coverslips (Marienfeld Superior) using ProLong Diamond (Thermo Fisher Scientific) for 24 hours to harden. STED microscopy was performed using an Abberior Expert Line microscope (Abberior). For the excitation of conjugated phalloidin and titin epitopes, 488 nm and 560 nm laser illumination sources were used, respectively. For STED imaging of the different titin epitopes labeled with STAR580 dye, a 775 nm depletion laser was utilized. Images were acquired with a Nikon CFI PL APO 100× (NA = 1.45) oil immersion objective coupled with avalanche photodiode detectors with spectral detection capabilities. Deconvolution of the recorded STED images was performed with Huygens Professional software (SVI) using the theoretical point spread function (PSF) of the imaging objective. Fluorescence intensity plot profiles were generated with Fiji (based on ImageJ, version 1.52). Plot profiles were fitted with Gaussian curves to determine the epitope peak position, height, and full width at half maximum (FWHM) using Fityk 1.3.0 software. A-band width was determined from the MIR epitope positions across the Z-disk. Fluorescence intensity normalization of signals of the A170 titin epitope was performed on carefully preselected plot profiles. For this purpose, background intensity–corrected plot profiles were collected, with averaging across a thick line to compensate for labeling inhomogeneity along the epitope lines. Plot profiles were discarded in case the intensity fluctuation of either the MIR or the A170 epitopes across the M-line exceeded 20% of the average intensity of the respective epitope. Fluorescence intensity was calculated from the peak height of the fitted Gaussians.

### Statistics.

Statistical analysis was performed with GraphPad Prism 8 (GraphPad Software). Continuous-variable statistical data are shown as the mean ± SEM unless stated otherwise. Differences between groups were considered to be statistically significant at a probability value of *P* < 0.05. A 2-tailed Student’s *t* test was used when comparing the means of 2 groups, and Welch’s correction was applied in the case of unequal variances between the 2 groups. Multiple comparisons were performed with ANOVA. Normality of statistical distribution was checked with the Shapiro-Wilk test. A Mann-Whitney *U*, Kruskal-Wallis, or ANOVA test was used for statistical comparisons of data for which normal distribution could not be assumed. In order to increase the statistical power of the tests, an equal or close-to-equal sample size was applied within independent groups. Linear regression analysis was performed to fit and compare sarcomere length–dependent epitope distance and fluorescence intensity data obtained from super-resolution microscopy. Sample randomization as well as blinding of the investigator were applied for analysis of the super-resolution microscopy images. For detailed statistical information, see the Supplemental Tables 1–16.

### Study approval.

The sample collection procedure and experiments were reviewed and approved by institutional and national ethics committees (Semmelweis University Regional and Institutional Committee of Science and Research Ethics, Budapest 1091, Hungary; permission nos. ETT TUKEB 7891/2012/EKU [119/ PI/12.], TUKEB 73/2005, and IV/10161-1/2020/EKU). Written informed consent was obtained from the patients prior to sample collection, in accordance with the Declaration of Helsinki.

### Data availability.

All data presented in this work are available in the associated Supplemental Supporting Data Values file. The NGS data have been deposited in the European Genome-Phenome Archive (EGA) (https://ega-archive.org) under the title “Next-generation sequencing on cardiac samples in Hungarian patients of dilated cardiomyopathy” (accession no. EGA50000000043).

## Author contributions

DK and HT designed research studies, conducted experiments, acquired data, analyzed data, and wrote the manuscript. The order of the co–first authors’ names was determined on the basis of the amount of experiments and analyses conducted. BK conducted STED experiments, acquired and analyzed STED data. GT acquired data and analyzed STED data. PD analyzed STED data. AAS acquired patient information data. MP, IH, and BS provided patient samples. SL provided antibodies. TR acquired patient information data and provided patient samples. AG, GB, and CB acquired and analyzed NGS data. BM designed research studies and provided patient samples. MSZK designed research studies, conducted experiments, acquired data, analyzed data, and wrote the manuscript.

## Supplementary Material

Supplemental data

Supplemental table 1

Supplemental table 2

Supplemental table 3

Supplemental table 4

Supplemental table 5

Supplemental table 6

Supporting data values

## Figures and Tables

**Table 1:**
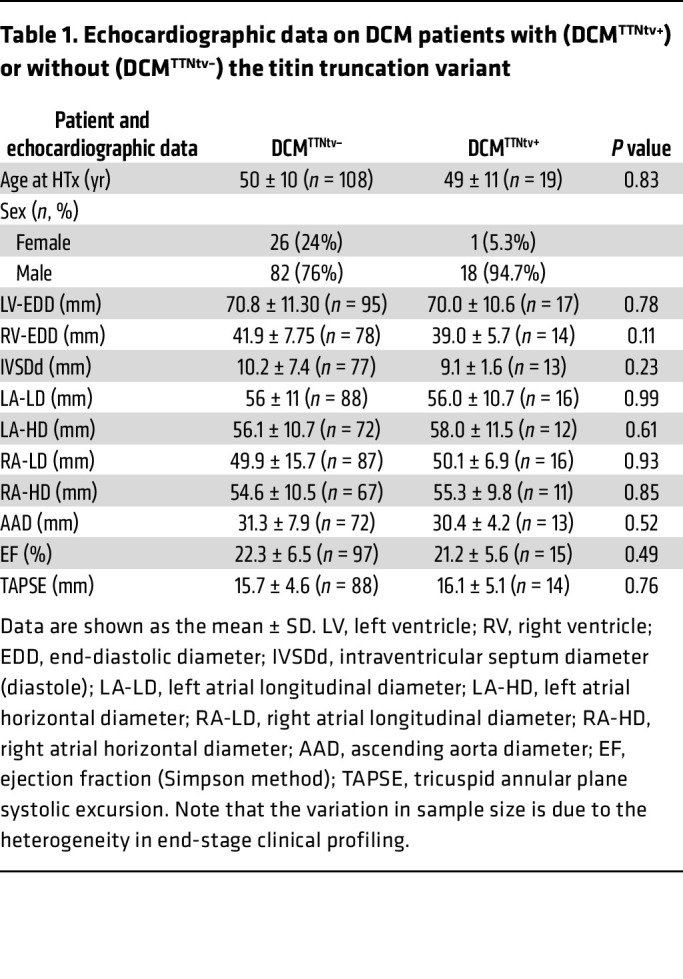
Echocardiographic data on DCM patients with (DCM^TTNtv+^) or without (DCM^TTNtv–^) the titin truncation variant

## Version 1 11/14/2023

In-Press Preview

## Version 2 01/16/2024

Electronic publication
