# Supplementary material for: Truncated titin is structurally integrated into the human dilated cardiomyopathic sarcomere
Source: J Clin Invest. 2024 Jan 16;134(2):e169753. doi: 10.1172/JCI169753 (PMC10763722; doi:10.1172/JCI169753)
Supplement: Supplemental data [file jci-134-169753-s011.pdf]

Supplementary information

Truncated titin is integrated into the human dilated cardiomyopathic sarcomere

Dalma Kellermayer[1,2,3], Hedvig Tordai[2], Balázs Kiss[2], György Török[2], Péter Dániel[2], Alex Ali Sayour[1], Miklós Pólos[1], István Hartyánszky[1], Bálint Szilveszter[1], Siegfried Labeit[4], Tamás Radovits[1], Ambrus Gángó[3], Gábor Bedics[3], Csaba Bödör[3], Béla Merkely[1] and Miklós Kellermayer[2*]

[1]Heart and Vascular Center, Semmelweis University, Budapest, Hungary
[2]Department of Biophysics and Radiation Biology, Semmelweis University, Budapest, Hungary
[3]1st Department of Pathology and Experimental Cancer Research, Semmelweis University, Budapest, Hungary
[4] DZHK Partnersite Mannheim-Heidelberg, Medical Faculty Mannheim, University of Heidelberg, Mannheim, Germany

Antibody information

Detailed information about the anti-titin antibodies can be obtained from Professor Siegfried Labeit (labeit@medma.de). The antigen used for generating the M8M10 antibody is specified below. The M8M10 anti-titin antibody is also available via Myomedix as TTN-9 (Myomedix Ltd., Biengarten 36, 69151 Neckargemuend, Germany, http://www.myomedix.com).

Expressed human titin antigen, based on Uniprot accession no Q8WZ42:
`*: natural endogenous stop codon in gene`
`Yellow: highlighted IG domain hydrophobic core used for counting IG domains.`

Anti-M8-M9-M10 C-terminal titin Antigen:
MSEAKSQEKLALKEEASKVLISEEVKKSAATSLEKSIVHEEITKTSQASE
EVRTHAEIKAFSTQMSINEGQRLVLKANIAGATDVKWVLNGVELTNSEEY
RYGVSGSDQTLTIKQASHRDEGILTCISKTKEGIVKCQYDLTLSKELSDA
PAFISQPRSQN*INEGQNVLFTCEISGEPSPEIEWFKNNLPISISSNVSIS*
*RSRNVYSLEIR*NASVSDSGKYTIKAKNFRGQCSATASLMVLRIPPKIEAL
PSDISIDEGKVLTVACAFTGEPTPEVTWSCGGRKIHSQEQGRFHIENTDD
LTTLIIMDVQKQDGGLYTLSLGNEFGSDSATVNIHIRSI*

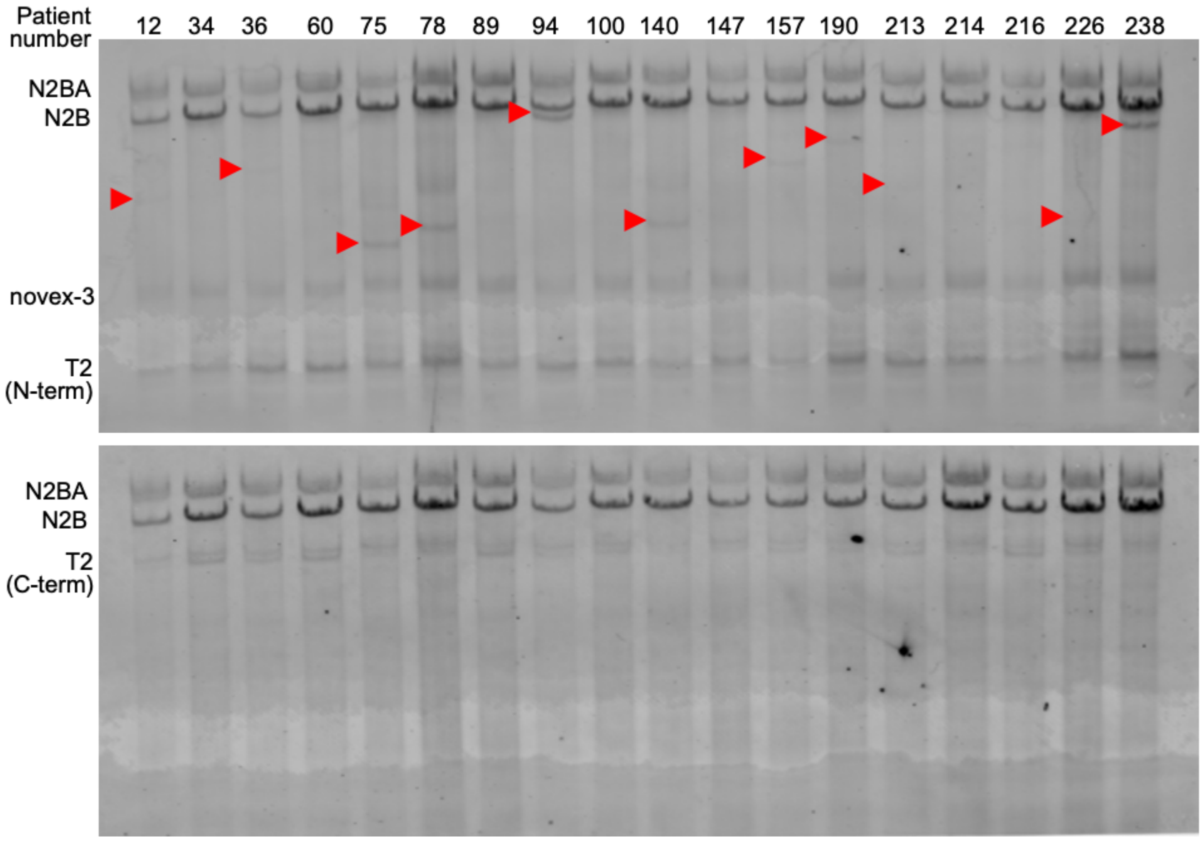

Figure S1. Western blot analysis on 18 of the DCM^{TTNtv+} samples differentiated the truncated titin from T2. Upper and lower panels show the samples labeled with the T12 and M8M10 anti-titin antibodies, respectively. Red arrows indicate the gel bands corresponding to truncated titin. Note that the penetrance of the truncated titin is uneven, and the truncated titin was not detectable in all TTNtv+ DCM samples. However, in samples #190, #213 and #226 the Western blot analysis detected truncated titin protein, even though the gel analysis (SYPRO Ruby staining) failed to do so (see **Supplementary Table S6**). Densitometric analysis of the Western blot and its comparison with the gel data are shown in **Table S16**.

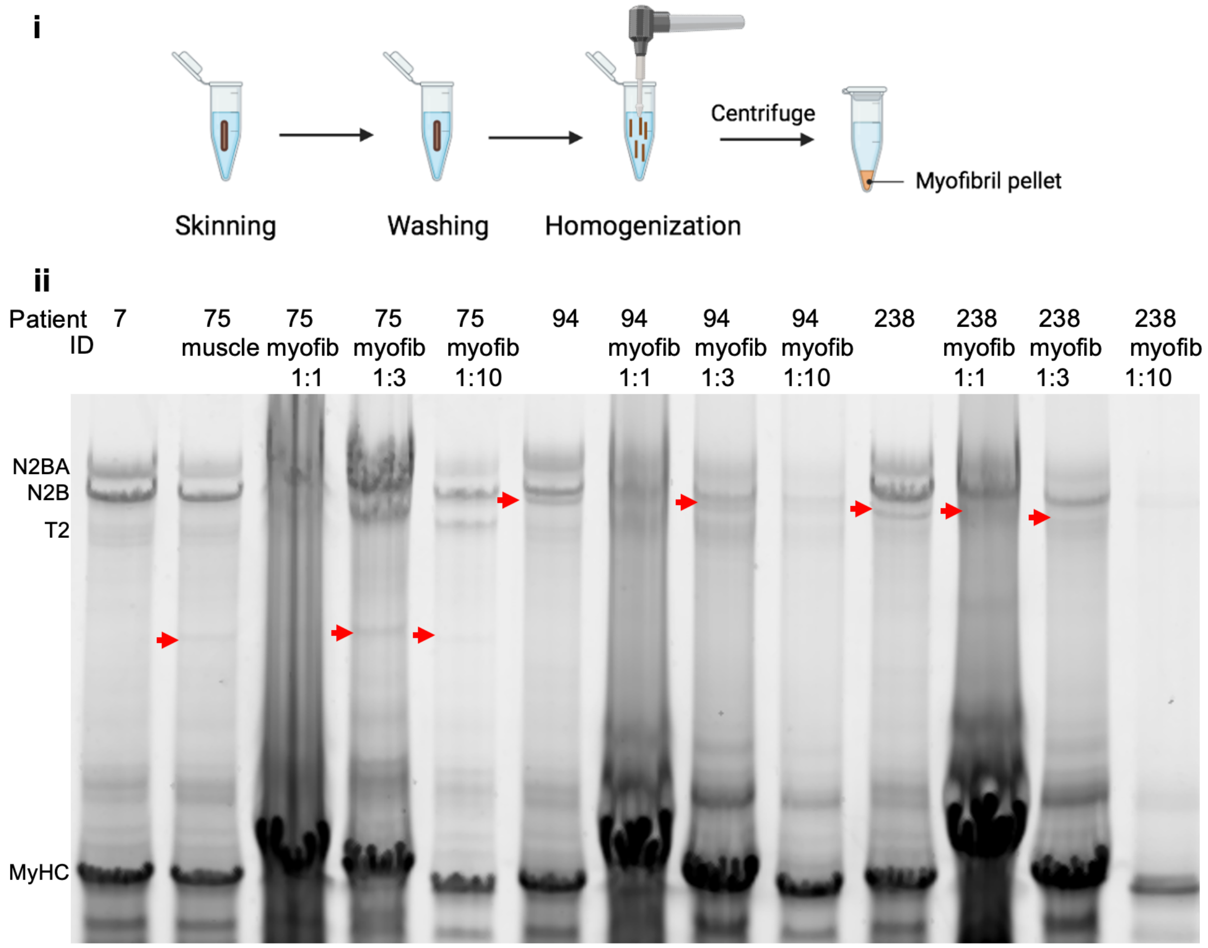

Figure S2A. Gel electrophoresis of washed myofibril samples. i. Schematic figure of washed myofibril suspension preparation (figure created with BioRender). **ii.** SDS-PAGE of TTNtv- (patient #7) and TTNtv+ (patients #75, 94 and 238) myofibrils. Washed myofibrils from TTNtv+ myocardial samples contain the truncated protein, indicating that the truncated titin is present in the sarcomere. 1:1, 1:3 and 1:10 myofibril:urea buffer dilutions were tested.

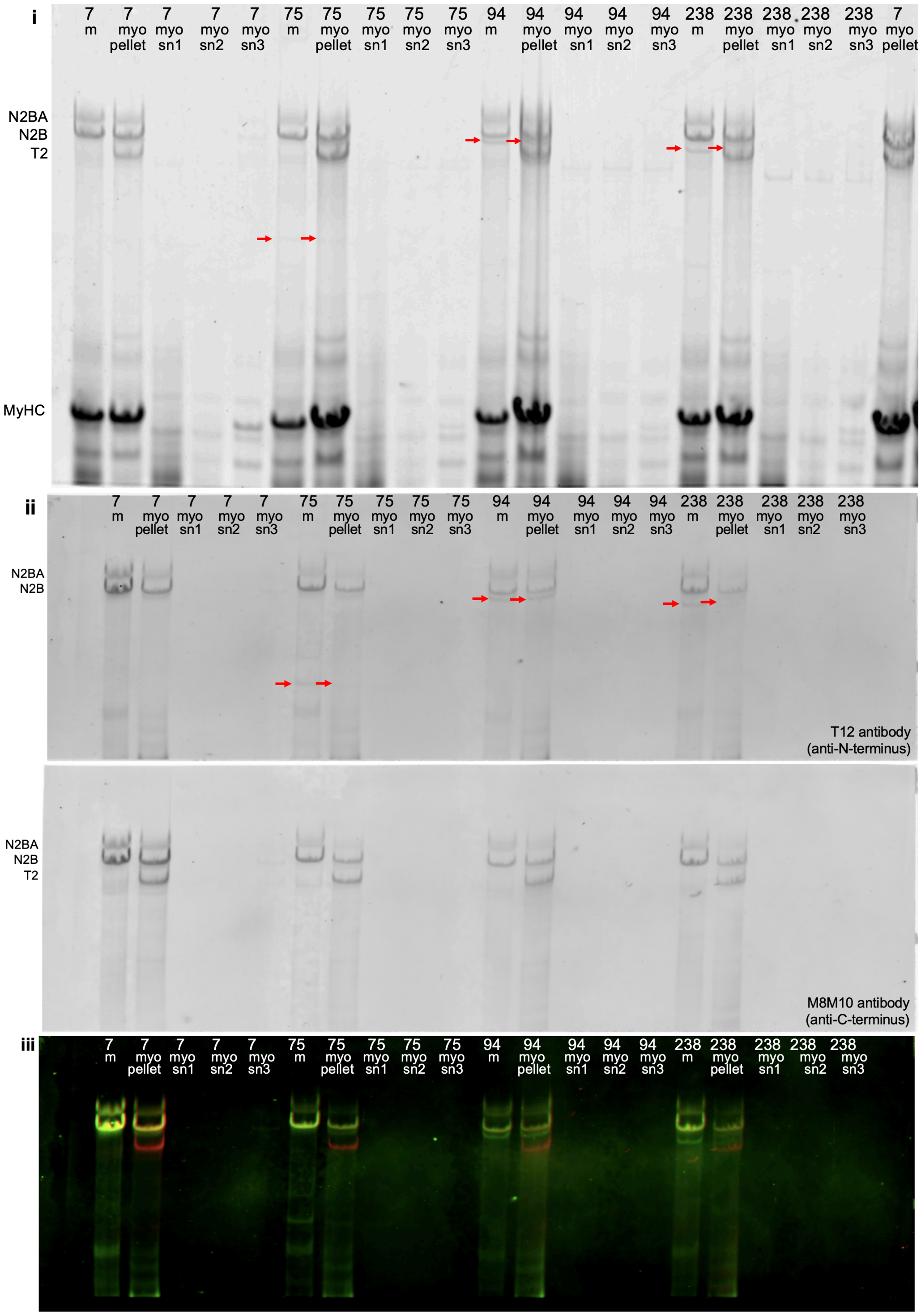

Figure S2B. Proteomics of washed myofibrils and corresponding supernatants. i. 1% agarose-PAGE of TTNtv- (patient #7) and TTNtv+ (patients #75, 94 and #238) myofibrils and their supernatants obtained in three subsequent washes. Supernatants were concentrated (Amicon Ultra-0.5 Filter, Merck Millipore, Burlington, MA) following each washing and centrifugation (2500 rpm, room temperature) step. The "pellet" lanes contain myofibril pellet:urea buffer ratios of 1:3, except in the last lane where this ratio is 1:1. **ii.** Western blot of the samples shown in **i**, using anti-N-terminus (T12, top image) and anti-C-terminus (M8M10, bottom image) anti-titin antibodies. **iii.** Overlay of the Western blots shown in **ii**. Yellow-green and red colors correspond to the T12 and M8M10 antibodies, respectively. Lane labels: m: muscle; myo: myofibril; sn1, sn2, sn3: 1x, 2x, 3x wash supernatants.

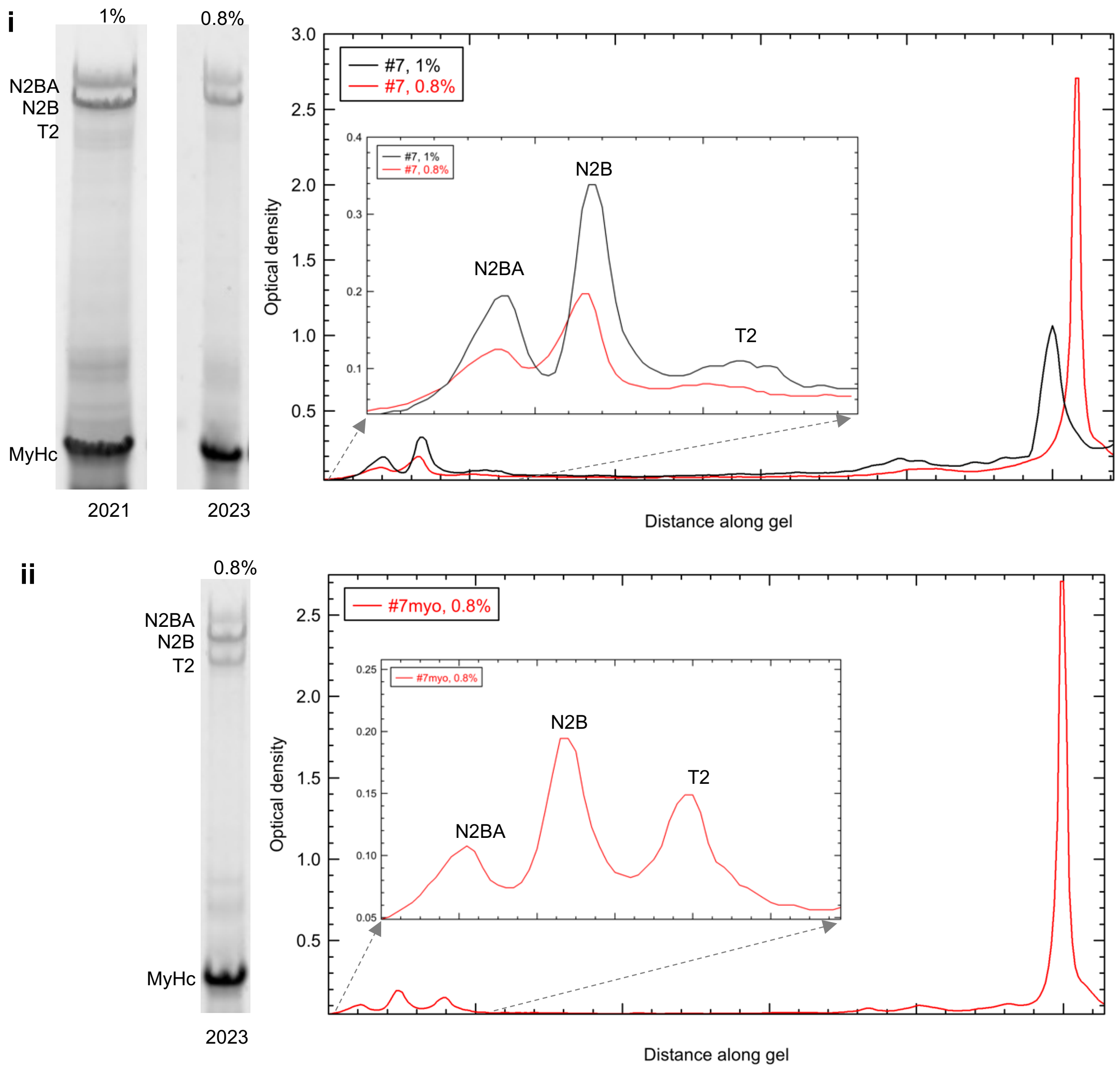

Figure S2C. High-resolution agarose gel electrophoretograms and corresponding optical density plot profiles of left ventricle (LV) muscle samples (**i**) and washed LV myofibrils (**ii**). The samples were from the TTN^{tv-} #7 patient. Two separate electrophoreses were carried out at different times (years 2021 and 2023) and slightly different gel densities (1% and 0.8%) for the muscle sample (**i**), but in this case only one electrophoresis was carried out for the washed myofibril sample (**ii**). Although small variations in the absolute optical densities of the protein bands may be observed, the peak ratios are conserved. The amount of the degradation product T2 is increased in myofibrils, which is a recurring finding (see below for the other samples as well). MyHc: myosin heavy chain.

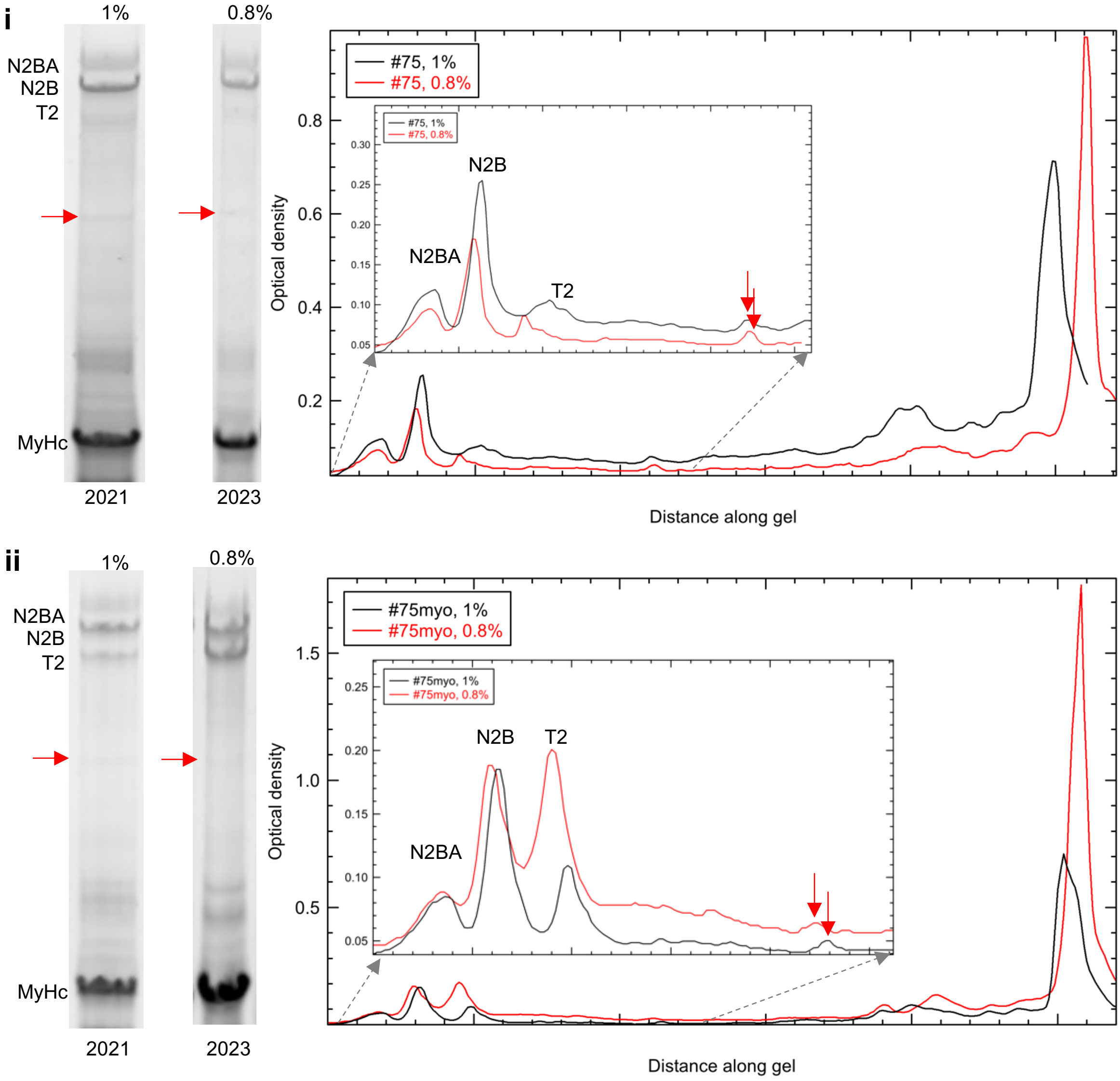

Figure S2D. High-resolution agarose gel electrophoretograms and corresponding optical density plot profiles of left ventricle (LV) muscle samples (**i**) and washed LV myofibrils (**ii**). The samples were from the TTN^{tv+} #75 patient. Two separate electrophoreses were carried out at different times (years 2021 and 2023) and slightly different gel densities (1% and 0.8%). Although small variations in the absolute optical densities of the protein bands may be observed, the peak ratios are conserved. The amount of the degradation product T2 is increased in myofibrils; however, even though the truncted protein has somewhat lower density (red arrow), it remains well detectable (see also Western blot results in **Figure S2B** above). Labels: MyHc: myosin heavy chain, red arrows: truncated titin.

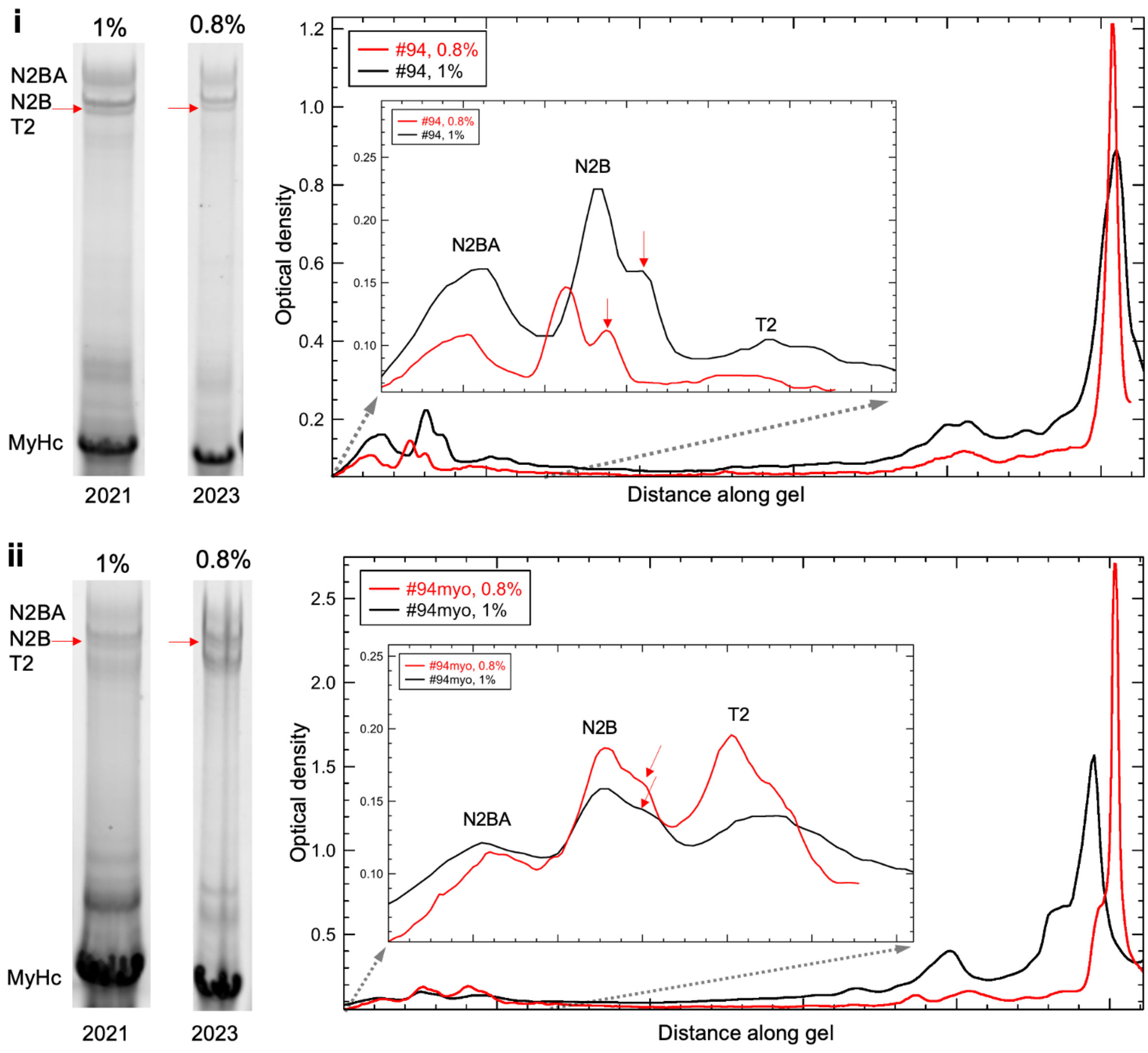

Figure S2E. High-resolution agarose gel electrophoretograms and corresponding optical density plot profiles of left ventricle (LV) muscle samples (**i**) and washed LV myofibrils (**ii**). The samples were from the TTN^{tv+} #94 patient. Two separate electrophoreses were carried out at different times (years 2021 and 2023) and slightly different gel densities (1% and 0.8%). Although small variations in the absolute optical densities of the protein bands may be observed, the peak ratios are conserved. The amount of the degradation product T2 is increased in myofibrils; however, even though the truncted protein has a lower density (red arrow), it remains well detectable (see also Western blot results in **Figure S2B** above). Labels: MyHc: myosin heavy chain, red arrows: truncated titin.

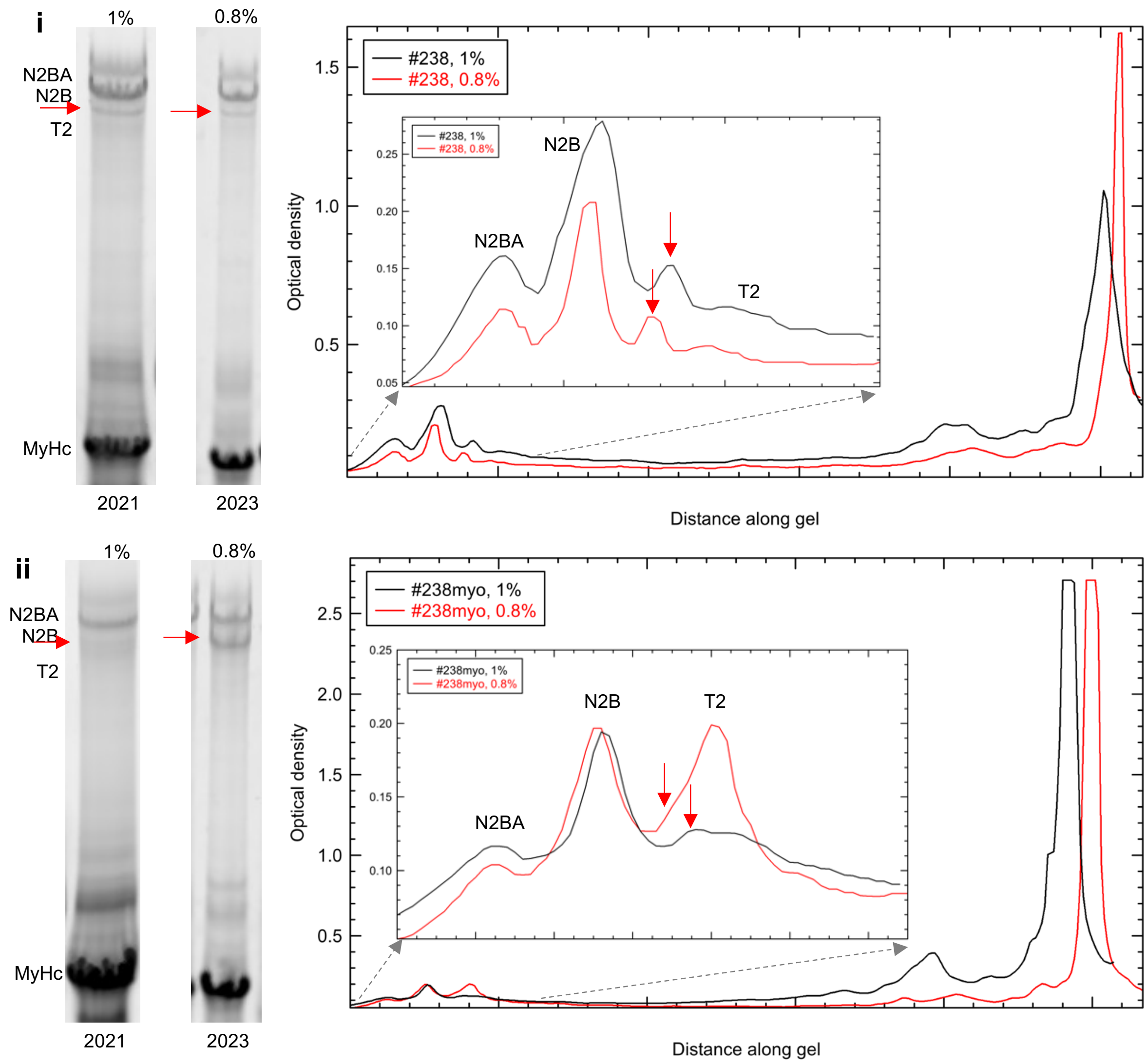

Figure S2F. High-resolution agarose gel electrophoretograms and corresponding optical density plot profiles of left ventricle (LV) muscle samples (**i**) and washed LV myofibrils (**ii**). The samples were from the TTN^{tv+} #238 patient. Two separate electrophoreses were carried out at different times (years 2021 and 2023) and slightly different gel densities (1% and 0.8%). Although small variations in the absolute optical densities of the protein bands may be observed, the peak ratios are conserved. The amount of the degradation product T2 is increased in myofibrils; however, even though the truncted protein has a lower density (red arrow), it remains well detectable (see also Western blot results in **Figure S2B** above). Labels: MyHc: myosin heavy chain, red arrows: truncated titin.

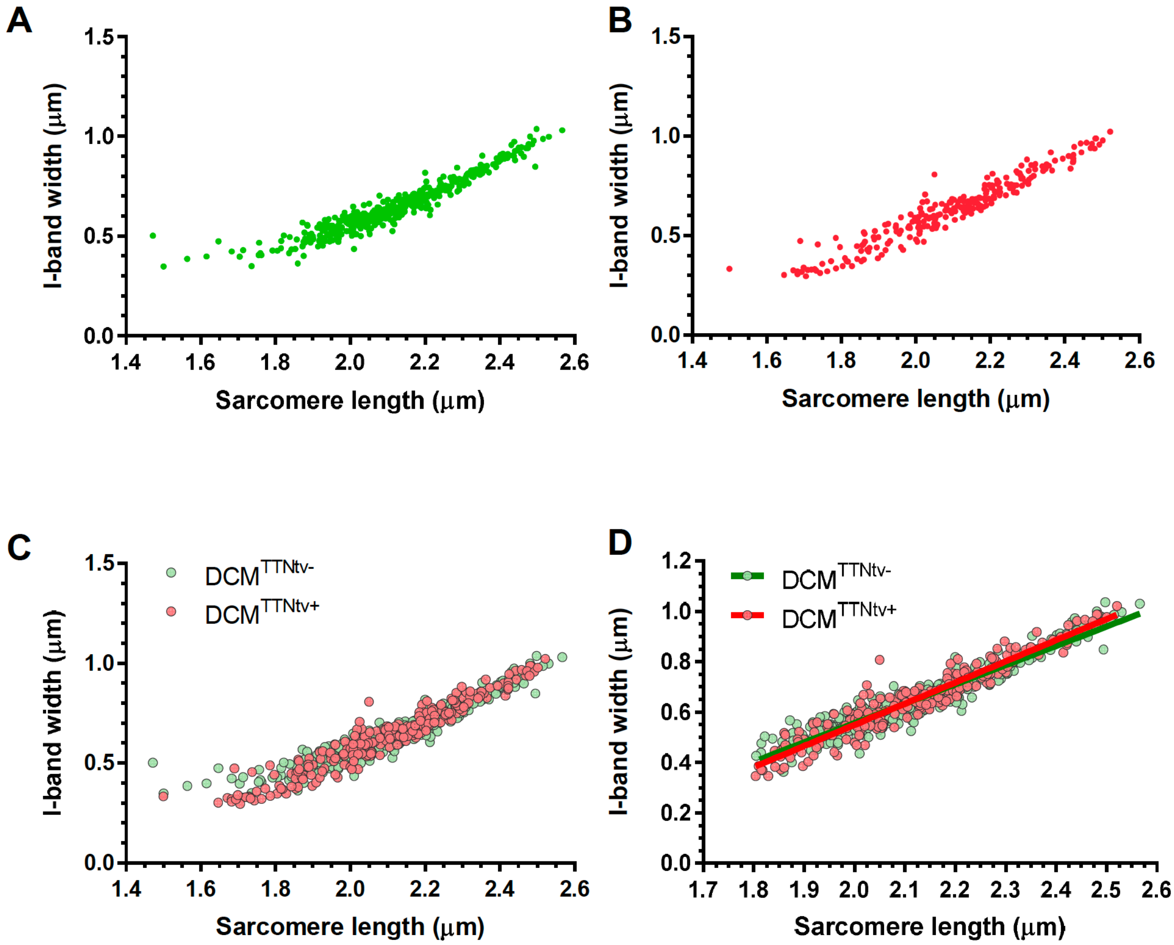

Figure S3. I-band titin length, measured with STED super-resolution microscopy, as a function of sarcomere length. A and **B,** I-band width as a function of the sarcomere length in DCM^{TTNtv-} and DCM^{TTNtv+} cardiac samples, respectively. I-band width was calculated as the distance between two consecutive MIR epitopes outside of the A-band (thus, not separated by an A170 epitope doublet). **C**, Overlaid data from DCM^{TTNtv-} and DCM^{TTNtv+} sarcomeres for comparison. **D**, Regression analysis of the I-band titin length in the 1.8-2.6 µm sarcomere length range, revealing significantly increased slope in DCM^{TTNtv+} fibers compared to that of the DCM^{TTNtv-}. The results complement those presented in **Figure 6E**, indicating that I-band titin in TTNtv+ sarcomeres extend more extensively than in TTNtv- ones. For statistics, see **Table S13**.

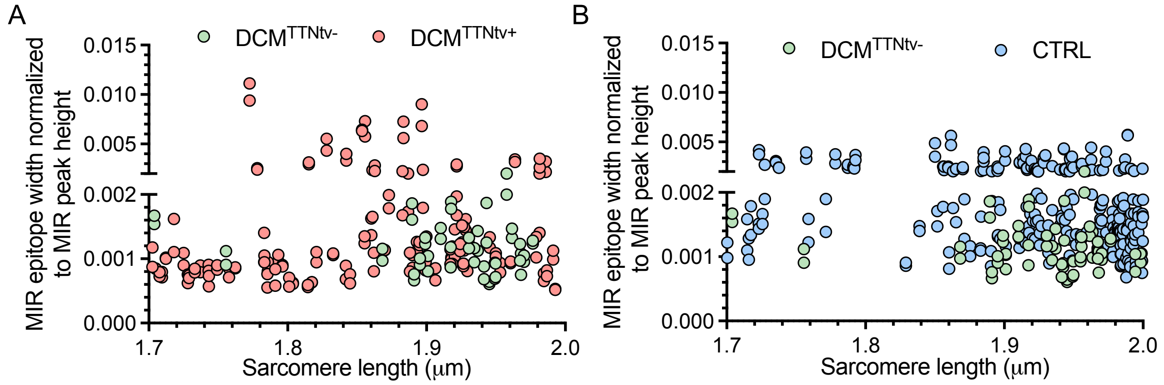

Figure S4. Normalized width distribution of the MIR epitopes in short sarcomeres with lengths between 1.7 and 2.0 μm. The comparisons are made between DCM^{TTNtv-} and DCM^{TTNtv+} (**A**) and between DCM^{TTNtv-} and normal control (CTRL, papillary muscle samples) (**B**).

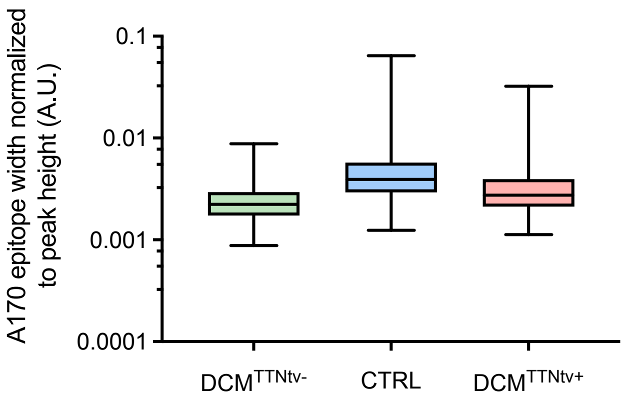

Figure S5. A170 anti-titin epitope width measured with STED super-resolution microscopy. FWHM values normalized to peak height of the A170 epitope of DCM^{TTNtv-} negative control (CTRL, papillary muscle) and DCM^{TTNtv+} sarcomeres with lengths between 1.8 and 2.6 µm. Block size reflects the standard error of the mean; the line across the box represents the median; whiskers indicate the minimum and maximum values, respectively. Note that only the full-length titin of the DCM^{TTNtv+} sarcomeres contain the A170 epitope. For statistics, see **Table S14.**

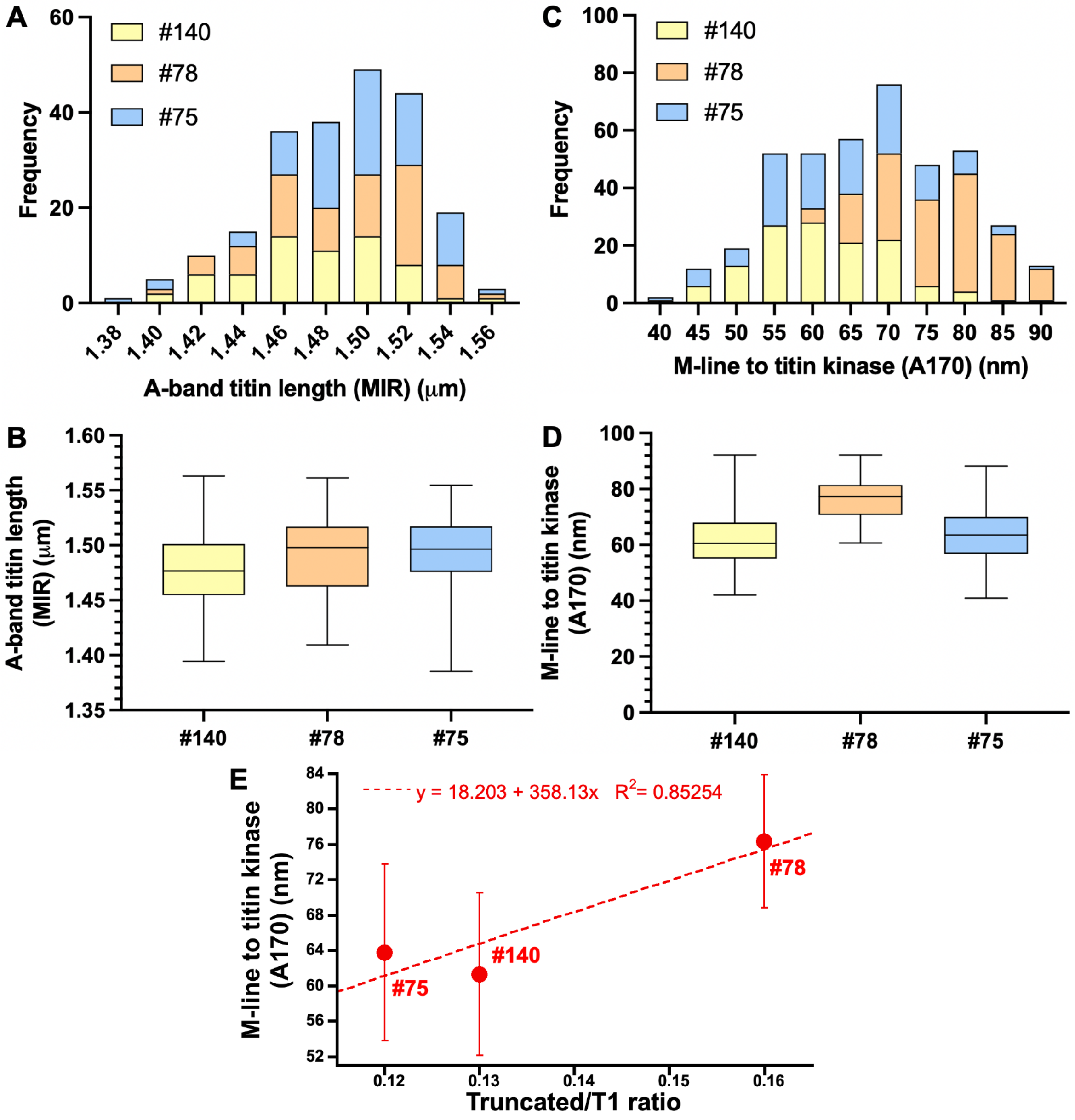

Figure S6. Sample-to-sample variaton of STED data in DCM^{TTNtv+} data. The location of the titin truncations in the individual samples are indicated in **Figure 1** of the main text. **A.** Histograms of the A-band titin length, measured via the MIR epitope-to-epitope distance. **B.** Histograms of the M-line to titin kinase distance, measured via the A170 epitope-to-epitope distance. **C.** Box plots of the A-band titin lengths in the different samples. **D.** Box plots of the M-line to titin kinase distance in the different samples. In the case of the MIR and A170 data, measurements in the sarcomere length range of 2.1-2.4 and 1.8-2.4 μm, respectively, were plotted and compared. Descriptive statistics of the data are shown in **Table S15**. The sample-to-sample variation in the A-band titin length is insignificant. By contrast, the M-line to titin kinase distance is significantly greater in sample #78 than in samples #75 and #140, in spite of their similar physical location along the titin gene. **E.** Mean M-line to titin kinase distance as a function of the truncated titin per T1 ratio. Error bars represent standard deviation. The truncated titin per T1 ratios, in samples where this could be quantified, are listed in **Table S6**.

Table S1. Blood test parameters of DCM patients before heart transplantation (see file DCM_MS_SI-table_1A.xlsx). ID: identification number of the transplanted patient, based on the sequence of the transplantation; Htx: heart transplantation; List type TX: transplantation, List type HU: high urgent; GFR: glomerular filtration rate; AST: aspartate transaminase, ALT: alanine transaminase; GGT: gamma-glutamyl transferase; ALP: alkaline phosphatase; WBC: white blood cell; CRP: c-reactive protein; LVAD: left ventricular assist device; ECMO: extracorporal membrane oxygenation.

Table S2. Extended parameters (echocardiography and N2BA/N2B ratio) of the DCM patients (both TTNtv+ and TTNtv-) the samples of whom were analyzed with STED microscopy (see file DCM_MS_SI-table_1B.xlsx).

Table S3. Bioinformatics overview of the pathogenic variants in the DCM samples (see file DCM_MS_SI-table_2.xlsx)**.**

Table S4. Sequence data of the pathogenic variants in the DCM cohort (see file DCM_MS_SI-table_3.xlsx)**.**

Table S5. Sequence data of the TTNtv+ samples (see file DCM_MS_SI-table_4A.xlsx)**.**

Table S6. Proteomic data of all analyzed samples (see file DCM_MS_SI-table_4B.xlsx)**.** The integrated densities (quantities) of the relevant protein bands and their ratios are indicated.

Table S7. Statistical analysis of the A-band titin length as a function of sarcomere length (analysis of data presented graphically in **Figure 6E**). The values missing from the regression analysis are the data points that fall below a sarcomere length of 1.8 µm. The slope has a unit of µm/µm, therefore it is dimensionless.

	TTNtv-	TTNtv+
**Best-fit values**		
**Slope**	**0.2357**	**0.1484**
Y-intercept	0.9703	1.156
X-intercept	-4.116	-7.788
1/slope	4.242	6.740
**Std. Error**		
Slope	0.007305	0.01179
Y-intercept	0.01560	0.02514
**95% Confidence Intervals**		
Slope	0.2214 to 0.2501	0.1252 to 0.1715
Y-intercept	0.9397 to 1.001	1.106 to 1.205
X-intercept	-4.521 to -3.758	-9.624 to -6.448
**Goodness of Fit**		
R squared	0.5414	0.2597
Sy.x	0.03461	0.04103
**Is slope significantly non-zero?**		
F	1041	158.2
DFn, DFd	1, 882	1, 451
P value	<0.0001	<0.0001
Deviation from zero?	Significant	Significant
**Equation**	Y = 0.2357*X + 0.9703	Y = 0.1484*X + 1.156
**Data**		
Number of X values	917	1416
Maximum number of Y replicates	1	1
Total number of values	884	453

Table S8. Statistical comparison of A-band titin length as a function of sarcomere length in negative control and DCM^{TTNtv-} samples. A-band titin length was measured as the distance between MIR epitopes across the A-band. There is no significant difference in the slopes of the linear fit, but there is a significant, ~44 nm difference in the Y-axis intercepts (corresponding graph is shown in **Figure 6F**).

	**DCM^{TTNtv-}**	**CTRL**
**Best-fit values**		
Slope	0.2359	0.2404
Y-intercept	0.9699	1.014
X-intercept	-4.111	-4.217
1/slope	4.238	4.160
**Std. Error**		
Slope	0.007284	0.007781
Y-intercept	0.01555	0.01671
**95% Confidence Intervals**		
Slope	0.2216 to 0.2502	0.2251 to 0.2557
Y-intercept	0.9394 to 1.000	0.9810 to 1.047
X-intercept	-4.514 to -3.754	-4.649 to -3.837
**Goodness of Fit**		
R squared	0.5433	0.5226
Sy.x	0.03453	0.03575
**Is slope significantly non-zero?**		
F	1049	954.5
DFn, DFd	1, 882	1, 872
P value	<0.0001	<0.0001
Deviation from zero?	Significant	Significant
**Equation**	Y = 0.2359*X + 0.9699	Y = 0.2404*X + 1.014
**Data**		
Number of X values	917	1854
Maximum number of Y replicates	1	1
Total number of values	884	874

Table S9. Statistical analysis of the M-band-to-A170 epitope distance as a function of sarcomere length (analysis of data graphically presented in **Figure 6H**). The values missing from the regression analysis are the data points that fall below a sarcomere length of 1.8 µm. The slope has a unit of nm/µm.

	TTNtv-	TTNtv+
**Best-fit values**		
**Slope**	**23.21**	**-14.11**
Y-intercept	16.81	97.00
X-intercept	-0.7243	6.874
1/slope	0.04309	-0.07087
**Std. Error**		
Slope	2.348	3.215
Y-intercept	5.015	6.852
**95% Confidence Intervals**		
Slope	18.60 to 27.82	-20.43 to -7.793
Y-intercept	6.966 to 26.65	83.53 to 110.5
X-intercept	-1.432 to -0.2506	5.404 to 10.73
**Goodness of Fit**		
R squared	0.1006	0.04115
Sy.x	11.24	11.34
**Is slope significantly non-zero?**		
F	97.69	19.27
DFn, DFd	1, 873	1, 449
P value	<0.0001	<0.0001
Deviation from zero?	Significant	Significant
**Equation**	Y = 23.21*X + 16.81	Y = -14.11*X + 97.00
**Data**		
Number of X values	911	1408
Maximum number of Y replicates	1	1
Total number of values	875	451

Table S10. Statistical comparison of M-line to titin kinase distance as a function of sarcomere length in negative control and DCM^{TTNtv-} samples. The distance data were obtained by halving the distance between vicinal A170 epitopes. The slope of the CTRL data is not significantly different from zero (corresponding graph is shown in **Figure 6I**.

	DCM^{TTNtv-}	CTRL
**Best-fit values**		
Slope	23.20	-1.802
Y-intercept	16.82	67.13
X-intercept	-0.7250	37.24
1/slope	0.04310	-0.5548
**Std. Error**		
Slope	2.345	3.572
Y-intercept	5.009	7.685
**95% Confidence Intervals**		
Slope	18.60 to 27.80	-8.813 to 5.208
Y-intercept	6.992 to 26.65	52.04 to 82.21
X-intercept	-1.432 to -0.2516	9.323 to +infinity
**Goodness of Fit**		
R squared	0.1008	0.0002936
Sy.x	11.23	16.52
**Is slope significantly non-zero?**		
F	97.90	0.2546
DFn, DFd	1, 873	1, 867
P value	<0.0001	0.6140
Deviation from zero?	Significant	Not Significant
**Equation**	Y = 23.20*X + 16.82	Y = -1.802*X + 67.13
**Data**		
Number of X values	911	1847
Maximum number of Y replicates	1	1
Total number of values	875	869

The relative extensibility (E_{rel}) of different A-band titin segments (A-band section, titin kinase region) were calculated as

$$E_{rel} = \frac{S}{N}, \tag{1}$$

where S is the slope of the sarcomere-length-dependent epitope-to-epitope distance (see **Figures 6E-I** and **Tables S7-S10**), and N is a factor that normalizes to the slack lengths of the respective titin segments as

$$N = \frac{Slack_x}{Slack_{kinase}}, \tag{2}$$

where $Slack_x$ is the respective epitope-to-epitope distance at slack (1.8 µm sarcomere length) and $Slack_{kinase}$ is the distance of the M-line to the A170 epitope at a sarcomere length of 1.8 µm. The calculated E_{rel} values for the A-band section and kinase region of titin in DCM^{TTNtv-} were 10 nm/µm and 23 nm/µm, respectively.

Table S11. Statistical analysis of MIR epitope intensity-profile width normalized to peak height of the MIR epitope (analysis of data presented graphically in **Figures 7A and S4A**). Epitope width is the full width at half maximum (FWHM) intensity. Data were compared by one-way ANOVA. ANOVA summary (**top**) and multiple comparison (**bottom**) tables are shown. Note that in this analysis groups A, B and C refer to DCM^{TTNtv-}, CTRL and DCM^{TTNtv+}, respectively. Data are distributed non-normally in each dataset. Even though the mean MIR width is largest in the DCM^{TTNtv+} sample and is significantly greater than that in DCM^{TTNtv-}, the difference is not significant with respect to CTRL. We attribute this finding to systematic differences in the properties and handling of the negative control tissue sample (papillary muscle, surgical removal, non-standard fixation and incubation procedures).

**Table Analyzed**	**MIR FWHM (<20% diff in peak height)**				
Data sets analyzed	A-C				
**ANOVA summary**					
F	10.95				
P value	<0.0001				
P value summary	****				
Significant diff. among means (P < 0.05)?	Yes				
R square	0.01486				
**Brown-Forsythe test**					
F (DFn, DFd)	12.78 (2, 1451)				
P value	<0.0001				
P value summary	****				
Are SDs significantly different (P < 0.05)?	Yes				
**Bartlett's test**					
Bartlett's statistic (corrected)	1809				
P value	<0.0001				
P value summary	****				
Are SDs significantly different (P < 0.05)?	Yes				
**ANOVA table**	**SS**	**DF**	**MS**	**F (DFn, DFd)**	**P value**
Treatment (between columns)	0.0001378	2	6.892e-005	F (2, 1451) = 10.95	P<0.0001
Residual (within columns)	0.009136	1451	6.296e-006		
Total	0.009273	1453			
Data summary					
Number of treatments (columns)	3				
Number of values (total)	1454				

Number of families	1							
Number of comparisons per family	3							
Alpha	0.05							
**Tukey's multiple comparisons test**	**Mean Diff.**	**95.00% CI of diff.**	**Significant?**	**Summary**	**Adjusted P Value**			
DCM^{TTNtv-} vs. CTRL	-0.0005394	-0.0009573 to -0.0001214	Yes	**	0.0071	A-B		
DCM^{TTNtv-} vs. DCM^{TTNtv+}	-0.0006654	-0.001012 to -0.0003185	Yes	****	<0.0001	A-C		
CTRL vs. DCM^{TTNtv+}	-0.0001260	-0.0005445 to 0.0002924	No	ns	0.7596	B-C		
**Test details**	**Mean 1**	**Mean 2**	**Mean Diff.**	**SE of diff.**	**n1**	**n2**	**q**	**DF**
DCM^{TTNtv-} vs. CTRL	0.001327	0.001867	-0.0005394	0.0001782	578	302	4.281	1451
DCM^{TTNtv-} vs. DCM^{TTNtv+}	0.001327	0.001993	-0.0006654	0.0001479	578	574	6.364	1451
CTRL vs. DCM^{TTNtv+}	0.001867	0.001993	-0.0001260	0.0001784	302	574	0.9993	1451

Table S12. Statistical analysis of STED microscopic measurements of A170 epitope integrated intensity normalized to the MIR epitope peak intensity (analysis of data presented graphically in **Figure 7B**). Epitope intensity was measured as the area under the curve in the intensity profile. Data were compared by one-way ANOVA. ANOVA summary (**top**) and multiple comparison (**bottom**) tables are shown.

**Table Analyzed**	**A170-to-MIR intensity**				
Data sets analyzed	A-C				
**ANOVA summary**					
F	336.2				
P value	<0.0001				
P value summary	****				
Significant diff. among means (P < 0.05)?	Yes				
R square	0.3474				
**Brown-Forsythe test**					
F (DFn, DFd)	11.56 (2, 1263)				
P value	<0.0001				
P value summary	****				
Are SDs significantly different (P < 0.05)?	Yes				
**Bartlett's test**					
Bartlett's statistic (corrected)	22.98				
P value	<0.0001				
P value summary	****				
Are SDs significantly different (P < 0.05)?	Yes				
**ANOVA table**	**SS**	**DF**	**MS**	**F (DFn, DFd)**	**P value**
Treatment (between columns)	1.922	2	0.9609	F (2, 1263) = 336.2	P<0.0001
Residual (within columns)	3.610	1263	0.002858		
Total	5.531	1265			
**Data summary**					
Number of treatments (columns)	3				
Number of values (total)	1266				

Number of families	1							
Number of comparisons per family	3							
Alpha	0.05							
**Tukey's multiple comparisons test**	**Mean Diff.**	**95.00% CI of diff.**	**Significant?**	**Summary**	**Adjusted P Value**			
TTNtv- vs. control	0.03447	0.02516 to 0.04379	Yes	****	<0.0001	A-B		
TTNtv- vs. TTNtv+	0.08815	0.08014 to 0.09617	Yes	****	<0.0001	A-C		
control vs. TTNtv+	0.05368	0.04432 to 0.06304	Yes	****	<0.0001	B-C		
**Test details**	**Mean 1**	**Mean 2**	**Mean Diff.**	**SE of diff.**	**n1**	**n2**	**q**	**DF**
TTNtv- vs. control	0.2646	0.2302	0.03447	0.003969	496	286	12.28	1263
TTNtv- vs. TTNtv+	0.2646	0.1765	0.08815	0.003416	496	484	36.50	1263
control vs. TTNtv+	0.2302	0.1765	0.05368	0.003987	286	484	19.04	1263

Table S13. Statistical analysis of I-band titin extension as a function of sarcomere length (analysis of data presented graphically in **Figure S3**). The values missing from the regression analysis are the data points that fall below a sarcomere length of 1.8 µm. The slope has a unit of µm/µm, therefore it is dimensionless. The two slopes are significantly different (p=0.0001).

	**TTNtv-**	**TTNtv+**
**Best-fit values ± SE**		
Slope	0.766 ± 0.01078	0.8396 ± 0.01723
Y-intercept	-0.9752 ± 0.02302	-1.13 ± 0.03673
X-intercept	1.273	1.345
1/slope	1.305	1.191
**95% Confidence Intervals**		
Slope	0.7448 to 0.7872	0.8057 to 0.8736
Y-intercept	-1.02 to -0.93	-1.202 to -1.057
X-intercept	1.248 to 1.297	1.312 to 1.376
**Goodness of Fit**		
R square	0.9195	0.9131
Sy.x	0.03605	0.04326
**Is slope significantly non-zero?**		
F	5051	2376
DFn, DFd	1, 442	1, 226
P value	<0.0001	<0.0001
Deviation from zero?	Significant	Significant
**Equation**	Y = 0.766*X - 0.9752	Y = 0.8396*X - 1.13
**Data**		
Number of X values	460	711
Maximum number of Y replicates	1	1
Total number of values	444	228

Table S14. Statistical analysis of STED microscopic measurements of normalized A170 epitope intensity-profile width (analysis of data presented graphically in **Figure S5).** Data were compared by one-way ANOVA. ANOVA summary (**top**) and multiple comparison (**bottom**) tables are shown.

**Table Analyzed**	**A170 FWHM (<20% diff in peak height)**				
Data sets analyzed	A-C				
**ANOVA summary**					
F	59.60				
P value	<0.0001				
P value summary	****				
Significant diff. among means (P < 0.05)?	Yes				
R squared	0.06795				
**Brown-Forsythe test**					
F (DFn, DFd)	30.43 (2, 1635)				
P value	<0.0001				
P value summary	****				
Are SDs significantly different (P < 0.05)?	Yes				
**Bartlett's test**					
Bartlett's statistic (corrected)	1372				
P value	<0.0001				
P value summary	****				
Are SDs significantly different (P < 0.05)?	Yes				
**ANOVA table**	**SS**	**DF**	**MS**	**F (DFn, DFd)**	**P value**
Treatment (between columns)	0.003661	2	0.001831	F (2, 1635) = 59.60	P<0.0001
Residual (within columns)	0.05022	1635	3.072e-005		
Total	0.05389	1637			
**Data summary**					
Number of treatments (columns)	3				
Number of values (total)	1638				

Number of families	1							
Number of comparisons per family	3							
Alpha	0.05							
**Tukey's multiple comparisons test**	**Mean Diff.**	**95.00% CI of diff.**	**Below threshold?**	**Summary**	**Adjusted P Value**			
DCM^{TTNtv-} vs. CTRL	-0.003565	-0.004338 to -0.002792	Yes	****	<0.0001	A-B		
DCM^{TTNtv-} vs. DCM^{TTNtv+}	-0.001591	-0.002422 to -0.0007607	Yes	****	<0.0001	A-C		
CTRL vs. DCM^{TTNtv+}	0.001974	0.001195 to 0.002753	Yes	****	<0.0001	B-C		
**Test details**	**Mean 1**	**Mean 2**	**Mean Diff.**	**SE of diff.**	**n1**	**n2**	**q**	**DF**
DCM^{TTNtv-} vs. CTRL	0.002512	0.006077	-0.003565	0.0003296	496	658	15.30	1635
DCM^{TTNtv-} vs. DCM^{TTNtv+}	0.002512	0.004103	-0.001591	0.0003541	496	484	6.355	1635
CTRL vs. DCM^{TTNtv+}	0.006077	0.004103	0.001974	0.0003319	658	484	8.411	1635

Table S15. Descriptive statistics of STED data from different DCM^{TTNtv+} samples. Histograms and box plots of the data are shown in **Figure S6**.

**Samples**	**#140**	**#78**	**#75**
**A-band titin length (MIR) (μm)**			
Number of values	63	75	82
Minimum	1.394	1.410	1.385
Maximum	1.563	1.561	1.555
Range	0.1685	0.1519	0.1695
Mean	1.476	1.491	1.494
Std. Deviation	0.03492	0.03421	0.03252
Std. Error of Mean	0.004400	0.003950	0.003591
**M-line to titin kinase (A170) (nm)**			
Number of values	130	157	124
Minimum	42.03	60.68	40.95
Maximum	92.17	92.20	88.24
Range	50.14	31.52	47.29
Mean	61.32	76.36	63.76
Std. Deviation	9.165	7.553	9.974
Std. Error of Mean	0.8038	0.6028	0.8957

Table S16. Statistical comparison of truncated titin quantities detected with gel electrophoresis and Western blotting. Upper part shows the densitometry results of the Western blot (**Figure S1**), lower part shows the comparison with the gel densitometry data.

Sample# (with TTNtv mutation)	N2BA	N2B	TTNtv	T1	TTNtv/T1
12	16723.296	14509.468	4402.376	31232.764	0.140953775
34					
36	15604.296	16700.004	4713.083	32304.3	0.14589646
60					
75	5505.669	14039.054	4608.648	19544.723	0.235800119
78	11600.761	17220.66	4903.134	28821.421	0.170121175
89					
94	13874.104	17729.761	9770.033	31603.865	0.309140448
100					
140	8119.225	17433.004	4864.941	25552.229	0.19039204
147					
157	11650.69	15531.418	3639.477	27182.108	0.133892375
190	10713.397	16345.468	3880.728	27058.865	0.143417989
213	10973.154	16728.711	1826.485	27701.865	0.065933647
214					
216					
226	10078.175	16343.418	2026.355	26421.593	0.076693143
238	11882.933	20879.66	7190.882	32762.593	0.21948452
**Sample# (with detectable TTNtv protein)**	**TTNtv/T1 (Western blot)**	**TTNtv/T1 (Gel electrophoresis)**			
12	0.140953775	0.185064935			
36	0.14589646	0.130541872			
75	0.235800119	0.122608079			
78	0.170121175	0.158974359			
94	0.309140448	0.333333333			
140	0.19039204	0.130387931			
157	0.133892375	0.152452026			
190	0.143417989				
213	0.065933647				
226	0.076693143				
238	0.21948452	0.272013949			
**Average**	0.166520517	0.185672061			
**StDev**	0.070279525	0.076670183			
**SQRT**	3.31662479	2.828427125			
**SEM**	0.021190074	0.027107003			
**p-value (t-test)**		0.579607013			
